# A mitochondria-related genes associated neuroblastoma signature - based on bulk and single-cell transcriptome sequencing data analysis, and experimental validation

**DOI:** 10.3389/fimmu.2024.1415736

**Published:** 2024-06-19

**Authors:** Chaoyu Wang, Jiaxiong Tan, Yan Jin, Zongyang Li, Jiaxing Yang, Yubin Jia, Yuren Xia, Baocheng Gong, Qiuping Dong, Qiang Zhao

**Affiliations:** ^1^ Department of Pediatric Oncology, Tianjin Medical University Cancer Institute & Hospital, National Clinical Research Center for Cancer, Tianjin's Clinical Research Center for Cancer, Key Laboratory of Cancer Prevention and Therapy, Tianjin, China; ^2^ Department of Tumor Cell Biology, Tianjin Medical University Cancer Institute and Hospital, National Clinical Research Center for Cancer, Tianjin's Clinical Research Center for Cancer, Key Laboratory of Cancer Prevention and Therapy, Tianjin, China

**Keywords:** neuroblastoma, mitochondria, signature, prognosis, tumor immune microenvironment, FEN1

## Abstract

**Background:**

Neuroblastoma (NB), characterized by its marked heterogeneity, is the most common extracranial solid tumor in children. The status and functionality of mitochondria are crucial in regulating NB cell behavior. While the significance of mitochondria-related genes (MRGs) in NB is still missing in key knowledge.

**Materials and methods:**

This study leverages consensus clustering and machine learning algorithms to construct and validate an MRGs-related signature in NB. Single-cell data analysis and experimental validation were employed to characterize the pivotal role of FEN1 within NB cells.

**Results:**

MRGs facilitated the classification of NB patients into 2 distinct clusters with considerable differences. The constructed MRGs-related signature and its quantitative indicators, mtScore and mtRisk, effectively characterize the MRGs-related patient clusters. Notably, the MRGs-related signature outperformed MYCN in predicting NB patient prognosis and was adept at representing the tumor microenvironment (TME), tumor cell stemness, and sensitivity to the chemotherapeutic agents Cisplatin, Topotecan, and Irinotecan. FEN1, identified as the most contributory gene within the MRGs-related signature, was found to play a crucial role in the communication between NB cells and the TME, and in the developmental trajectory of NB cells. Experimental validations confirmed FEN1’s significant influence on NB cell proliferation, apoptosis, cell cycle, and invasiveness.

**Conclusion:**

The MRGs-related signature developed in this study offers a novel predictive tool for assessing NB patient prognosis, immune infiltration, stemness, and chemotherapeutic sensitivity. Our findings unveil the critical function of FEN1 in NB, suggesting its potential as a therapeutic target.

## 1 Introduction

Neuroblastoma (NB), the most common extracranial solid tumor in children, originates from embryonic neural crest cells and accounting for 15% of all childhood cancer deaths (1, 2). Characterized by its marked heterogeneity, NB presents a varied clinical spectrum. Patients with low to intermediate-risk NB exhibit a survival rate exceeding 95%, with some cases even showing spontaneous regression without the need for therapeutic intervention; while the long-term survival rate for individuals with high-risk NB remains dismal, falling below 50% (3). There is also significant intratumor heterogeneity between cells within the same individual NB patient, and a hallmark feature of high-risk NB is the presence of multiple cell subsets (4). Therefore, individualized precision treatment is particularly important in NB.

NB is traditionally classified as an immunosuppressive “cold” tumor, characterized by low immunogenicity and a poor response to immunotherapeutic interventions (5, 6). While recent advancements in immunotherapy have significantly improved survival rates for several highly immunogenic adult solid tumors, the treatment efficacy for NB remains substantially challenged by its immunosuppressive microenvironment, with the majority of pediatric patients deriving minimal benefit from current immunotherapeutic approaches (7, 8). Consequently, identifying strategies to transform the immunosuppressive “cold” tumor into an immunostimulatory “hot” tumor, conducive to tumor immune microenvironment (TIME) activation, represents a critical and urgent task for enhancing the efficacy of immunotherapy in the clinical management of NB.

Mitochondria are increasingly recognized for their critical roles in the etiology and advancement of malignant tumors, acting through a plethora of mechanisms (9, 10). Their status and functionality are crucial in regulating tumor cell apoptosis, cell cycle progression, metabolic pathways, and so on (11, 12). The interaction between tumor cells and the tumor microenvironment (TME) is also modulated by mitochondrial dynamics, which extends to affecting the efficacy of immune cells within the TME, facilitating immune evasion, and contributing to the development of resistance to treatments (13, 14). The significance of mitochondria-related genes (MRGs) in malignancies, including but not limited to NB, is evident through their substantial impact on patient prognosis (15–17). Research into MRGs-related prognostic signatures in cancers such as lung adenocarcinoma, stomach adenocarcinoma, and breast cancer has shown promising results (18–20). However, the exploration of such prognostic models in NB is still absent, underscoring a critical gap in current knowledge and presenting a clear opportunity for groundbreaking contributions to personalized cancer therapy.

This study embarks on constructing a prognostic model for NB using MRGs through a series of bioinformatics methods and machine learning algorithms, aiming to categorize patients for more targeted clinical management and therapeutic strategies. This study delves into the application of the MRGs-related signature to delineate the TIME of NB patients, assessing tumor cell stemness, and evaluating chemotherapy drug sensitivity. This comprehensive approach seeks to enhance precision in patient classification, thereby facilitating clinical benefits. Moreover, through single-cell transcriptomic analysis and experimental validation, this research explores the significant role of FEN1, the most critical molecule within the MRGs-related signature, in NB, suggesting FEN1 as a potential therapeutic target and offering new avenues for treatment strategies.

## 2 Materials and methods

### 2.1 Data sources

The data of bulk RNA sequencing in GSE49710 (21) and single cell RNA sequencing in GSE137804 (22) were acquired from Gene Expression Omnibus (GEO, https://www.ncbi.nlm.nih.gov/geo/). The microarray data E-MTAB-8248 was obtained from ArrayExpress database (https://www.ebi.ac.uk/biostudies/arrayexpress) (23). The genomic data of NBL (neuroblastoma) project in TARGET (Therapeutically Applicable Research to Generate Effective Treatments) database was downloaded from the Genomic Data Commons (GDC, https://portal.gdc.cancer.gov) (24). The **Supplementary Table 1** presents the clinical baseline characteristics of the 4 datasets included in this study. The list of 2,030 MRGs was derived from the study by J. Chang, et al. (19).

### 2.2 Screening of differentially expressed genes

The “limma” package was used to screen differentially expressed genes (DEGs) in this study (25). Linear models were fitted using the lmFit function of the “limma” package and subsequently empirical Bayesian methods were applied using the eBayes function to stabilize the variance estimates. The P value < 0.05 and | log2 Fold change (FC) | > 1 were defined as the threshold for DEGs in this study.

### 2.3 Unsupervised clustering

The consensus clustering method and “ConsensusClusterPlus” package was performed to discover stable and consistent cluster structures in this study (26). One to nine clustering iterations were implemented in the datasets. In each iteration, the data are randomly split into subsets and then the K-means clustering algorithm is applied. Using the consistency matrix, the optimal number of clusters was determined by evaluating the consistency and stability of clusters under different cluster numbers (27).

### 2.4 Survival analysis

This study employed the “survival” package to conduct Kaplan-Meier (K-M) survival analysis (28, 29), a non-parametric method used to estimate the survival function from time-to-event data. And the “survminer” package was used to visualize survival estimates and generate the survival curves in this study (30).

### 2.5 Construction of prognostic signature

Prior to constructing a prognostic signature, this study initially employed K-M survival analysis and univariate Cox proportional hazards regression, both with OS as the endpoint, to screen for genes significantly associated with prognosis. In univariate COX regression models, the criteria were: P value < 0.05, and the 95% confidence interval (CI) of hazard ratio (HR) was consistently distributed ipsilateral to 1. Following the preliminary survival analysis, the machine learning algorithm Least Absolute Shrinkage and Selection Operator (LASSO) regression was utilized in GSE49710 dataset to determine the optimal number of genes and their respective coefficients for the prognostic model. The LASSO method was conducted using the “glmnet” package in R (31, 32). A score was obtained by linearly combining the mRNA expression levels of selected genes, each weighted by their respective coefficients derived from the LASSO regression analysis, which is termed the mitochondria-related risk score (mtScore).

### 2.6 Validation of prognostic signature

In order to evaluate the signature’s predictive capability across diverse patient populations, the same statistical method was applied to the GSE49710 dataset for internal validation and to two independent datasets E-MTAB-8248 and TARGET-NBL for external validation. K-M survival analysis, receiver operating characteristic (ROC) curve analysis and correlation analysis of key clinical features based on mtScore were all validated in the above different datasets. The area under the curve (AUC) value of ROC curves exceeded 0.70 was considered to be efficient prediction (33).

### 2.7 Analysis of immune infiltration

For the comprehensive evaluation of the immune cell infiltration within the TME, 4 prominent computational methods were employed: ESTIMATE (Estimation of STromal and Immune cells in MAlignant Tumour tissues using Expression data) (34), EPIC (Estimating the Proportions of Immune and Cancer cells) (35), MCPcounter (Microenvironment Cell Populations-counter) (36), and CIBERSORT (Cell-type Identification By Estimating Relative Subsets Of RNA Transcripts) (37). In addition, we also evaluated the infiltration of 28 kinds of immune cells provided by the study from Q. Jia, et al. (38). The infiltration abundance in TME of 28 different types of immune cells was calculated using the single-sample Gene Set Enrichment Analysis (ssGSEA) algorithm as described by D. A. Barbie, et al. (39).

### 2.8 Calculation of mRNA expression-based stemness index

To quantify the degree of NB cellular dedifferentiation, which is indicative of stemness characteristics within tumor samples, we employed the mRNA expression-based stemness index (mRNAsi). This index was calculated following the methodology developed by T. M. Malta, et al., leveraging a machine learning model predicated on the one-class logistic regression (OCLR) algorithm (40). The gene expression profile of GSE49710 used in this study was mapped against the stemness signature to calculate the mRNAsi score for each sample. The mRNAsi scores range from 0 to 1, with higher values indicating a closer resemblance to the pluripotent state, thereby suggesting higher tumor cell stemness.

### 2.9 Assessment of chemotherapeutic response

To evaluate the predictive value of mtRisk for drug sensitivity, the Genomics of Drug Sensitivity in Cancer (GDSC, https://www.cancerrxgene.org/) database was utilized to analyze the drug response of patients with varying mtRisk levels to 3 commonly used clinical drugs for NB patients (Cisplatin, Topotecan, and Irinotecan) (41). The half-maximal inhibitory concentration (IC50) serving as a gauge for drug potency computed by the he “DrugResponse” package (42).

### 2.10 Single-cell data pre-processing and single-cell communication analysis

In this study, single-cell RNA sequencing data from 16 samples, comprising 160,847 cells, were utilized for single-cell analysis. Initial data processing included quality control measures, notably the exclusion of cells characterized by an exceptionally low number of detected genes or elevated mitochondrial gene expression, followed by normalization and mitigation of batch effects. Subsequent data analysis involved dimensionality reduction using the Uniform Manifold Approximation and Projection (UMAP) algorithm, as implemented in the “Seurat” package in R (43). Cell types were annotated based on the cell markers recommended within the GSE137804 dataset.

To investigate the expression patterns of the FEN1 gene within tumor cells, all tumor cells were categorized into two groups based on the median expression level of FEN1: FEN1 high expression group (FEN1-High) and FEN1 low expression group (FEN1-Low). Inter-cellular communication was analyzed separately for FEN1-High and FEN1-Low tumor cells in relation to other cells within the TME. This analysis was facilitated using CellPhoneDB software (version 2.0; Wellcome Sanger Institute, Hinxton, Cambridge, UK) (44).

### 2.11 Single-cell pseudotime trajectory analysis

This study further explored the dynamic expression of FEN1 within the developmental trajectory of NB tumor cells. This study employed pseudotime trajectory analysis to simulate the continuum of cell differentiation states and to chart the progression of tumor cells from their origin to mature states. For this analysis, the “Monocle” package in R was utilized (45). Cells were ordered in a pseudotime sequence, an inferred temporal continuum that represents the maturation or progression of cells through a developmental pathway, based on their gene expression profiles. FEN1 expression levels were then quantitatively assessed across the pseudotime to elucidate the gene’s dynamic expression patterns during the development and differentiation of tumor cells. This differential expression analysis across pseudotime states aimed to identify significant changes in FEN1 expression, employing methods incorporated within the Monocle framework.

### 2.12 Overexpression and knockdown of FEN1 in NB cell

Human NB cell SH-SY5Y purchased from Meisen Chinese Tissue Culture Collections were cultured in Minimum Essential Medium/Ham’s F12 (MEM/F12) medium supplemented with 10% fetal bovine serum (FBS) and 1% penicillin-streptomycin (PS), under a humidified atmosphere containing 5% CO_2_ at 37°C to ensure healthy cell growth.

The overexpression (OE) and knockdown (KD) of FEN1 were achieved through infection procedure. Five distinct groups were structured in this study: Vector, FEN1, Scramble, sh-FEN1#1, and sh-FEN1#2. The pCDH-puro-FEN1 plasmid encoding human FEN1 was defined as OE of FEN1 and named “FEN1”. The empty pCDH-CMV-MCS-EF1-puro plasmid was defined as a negative control (NC) for OE (named “Vector”). Two short hairpin RNAs (shRNAs) targeting FEN1 was synthesized and inserted into the plasmid to generate the FEN1 KD vectors defines plsi-puro-FEN1–1 and plsi-puro-FEN1–2 (named “sh-FEN1#1” and “sh-FEN1#2”). The shRNAs sequences are shown in **Supplementary Table 2**. The NC for KD was constructed from a scrambled shRNA inserted into plsi-ctrl-puro plasmid named “Scramble”. The cloned plasmids and packaging plasmids (psPAX2 and pMD2-VSVG) were transfected into 293 T cells to synthesize the lentiviral particles. The NB cells were infected with the collected lentiviral particles. The total RNA and total protein of the infected NB cells were collected, and the efficiency of FEN1 OE and KD was verified by quantitative real-time PCR (qRT-PCR) and Western blot analysis.

### 2.13 Cell counting kit-8 assay

The NB cells were seeded in 96-well plates and cultured at 5% CO_2_, 37°C atmospheres. The incubation was continued for 3 hours after adding 10 μL of cell counting kit-8 (CCK-8) solution (CA1210, Solarbio, China) to each well. The absorbance at 450 nm was measured at four distinct time points: 0-, 24-, 48-, and 72-hours post-adherence, using a microplate reader. The relative cell proliferation activity was calculated according to the following formula:


$$\text{Relative proliferation}=\left({\mathrm{OD}}_{450mm}^{OE\;or\;KD}-{\mathrm{OD}}_{450mm}^{blank}\right)/\left({\mathrm{OD}}_{450mm}^{NC}-{\mathrm{OD}}_{450mm}^{blank}\right)$$

### 2.14 Plate cloning assay

The single-cell suspensions were prepared using 0.25% trypsin-EDTA solution. The NB cells were seeded in 6-well plates at a density of 1,000 cells per well. After one week of culture, colonies were fixed with 4% paraformaldehyde for 15 minutes at room temperature and subsequently stained with 0.1% crystal violet for 15 minutes. Excess stain was removed by washing the plates with distilled water, and the plates were allowed to air dry. Colonies consisting of more than 50 cells were counted manually under a light microscope.

### 2.15 Mitochondrial membrane potential ΔΨm assay with JC-1

The mitochondrial membrane potential (MMP) is a critical parameter in the regulation of cell apoptosis, serving as a key indicator of cell health (46). The decline of MMP is a hallmark event in the early stage of apoptosis. The JC-1, an ideal fluorescent probe widely used to detect the MMP ΔΨm (47), was applied as an indicator of apoptosis in this study (C2003, Beyotime, China). In detail, each of the 5 groups of NB cells were trypsinized, collected, and washed twice with cold phosphate-buffered saline (PBS). Cells were resuspended in 500 µL of PBS and subsequently mixed with 500 µL of JC-1 staining solution. The mixture was then incubated for 20 minutes at 37°C in the dark to allow for staining. After incubation, cells were washed twice with dye buffer and immediately analyzed by flow cytometry. For the detection of JC-1 monomers, the analysis conducted through the FITC channel. Conversely, the assessment of JC-1 aggregates, was performed with the PE channel for detection. A minimum of 20,000 events were recorded for each sample.

### 2.16 Cell cycle analysis

The NB cells in 5 groups were harvested and washed twice with cold PBS. Cells were then fixed in 70% ethanol at 4°C overnight. After fixation, cells were washed with PBS and then resuspended in 100 µL of RNase A solution (CA1510, Solarbio, China) and incubated at 37°C for 30 minutes. The cells were stained with propidium iodide (PI) (CA1510, Solarbio, China) for 30 minutes at 4°C in the dark. For the analysis of DNA content, the emitted fluorescence of PI-stained cells was detected in the PE channel using a flow cytometer. Data acquisition was performed for at least 20,000 cells per sample to ensure statistical relevance. The Dean-Jett-Fox model, a built-in algorithm within FlowJo, was employed to fit the DNA content histogram and quantitatively assess the proportions of cells in G0/G1, S, and G2/M phases of the cell cycle (48).

### 2.17 Transwell invasion assay

The invasive potential of the NB cells was assessed using Transwell permeable supports with 8.0 µm pore polystyrene membrane inserts. The upper surface of the insert was coated with 50 µL of Matrigel at a concentration of 2 mg/mL and allowed to solidify at 37°C incubator for 1 hour to form a thin layer of matrix barrier mimicking the extracellular matrix. Then, 1x10^5^ cells in 200 µL of serum-free medium were placed into the upper chamber, and 600 µL of medium containing 10% FBS was added to the lower chamber as a chemoattractant. After 48 hours of incubation at 37°C, non-invading cells on the upper surface of the membrane were gently removed with a cotton swab. Cells that had invaded through the Matrigel and reached the lower surface of the membrane were fixed with 4% paraformaldehyde, stained with 0.1% crystal violet, and counted under a light microscope in five randomly selected fields per well.

### 2.18 Statistical analysis

Continuous variables were expressed as mean ± standard deviation (SD). For comparisons between two groups, the Student’s t-test was employed. Categorical variables were presented as numbers (percentages) and analyzed using the Chi-square test. Correlations between continuous variables were evaluated using Pearson’s correlation coefficient. The P value < 0.05 was considered statistically significant for all tests. All experiment were performed in triplicate.

The bioinformatics analysis was carried out using R software (version 4.3.3; R Foundation for Statistical Computing, Vienna, Austria). Post-acquisition flow cytometry data processing was conducted using FlowJo (version 10.8.1; BD Biosciences, San Jose, California USA). Part of the statistical analysis and the generation of corresponding figures were performed with GraphPad Prism (version 9.0; GraphPad Software, San Diego, California USA). Image processing and assembly tasks were accomplished using Adobe Photoshop 2023 and Adobe Illustrator 2023 (Adobe Systems Incorporated, San Jose, California USA).

## 3 Results

### 3.1 MRGs-based clustering of NB patients into 2 distinct clusters with unique differences

**Figure 1:**
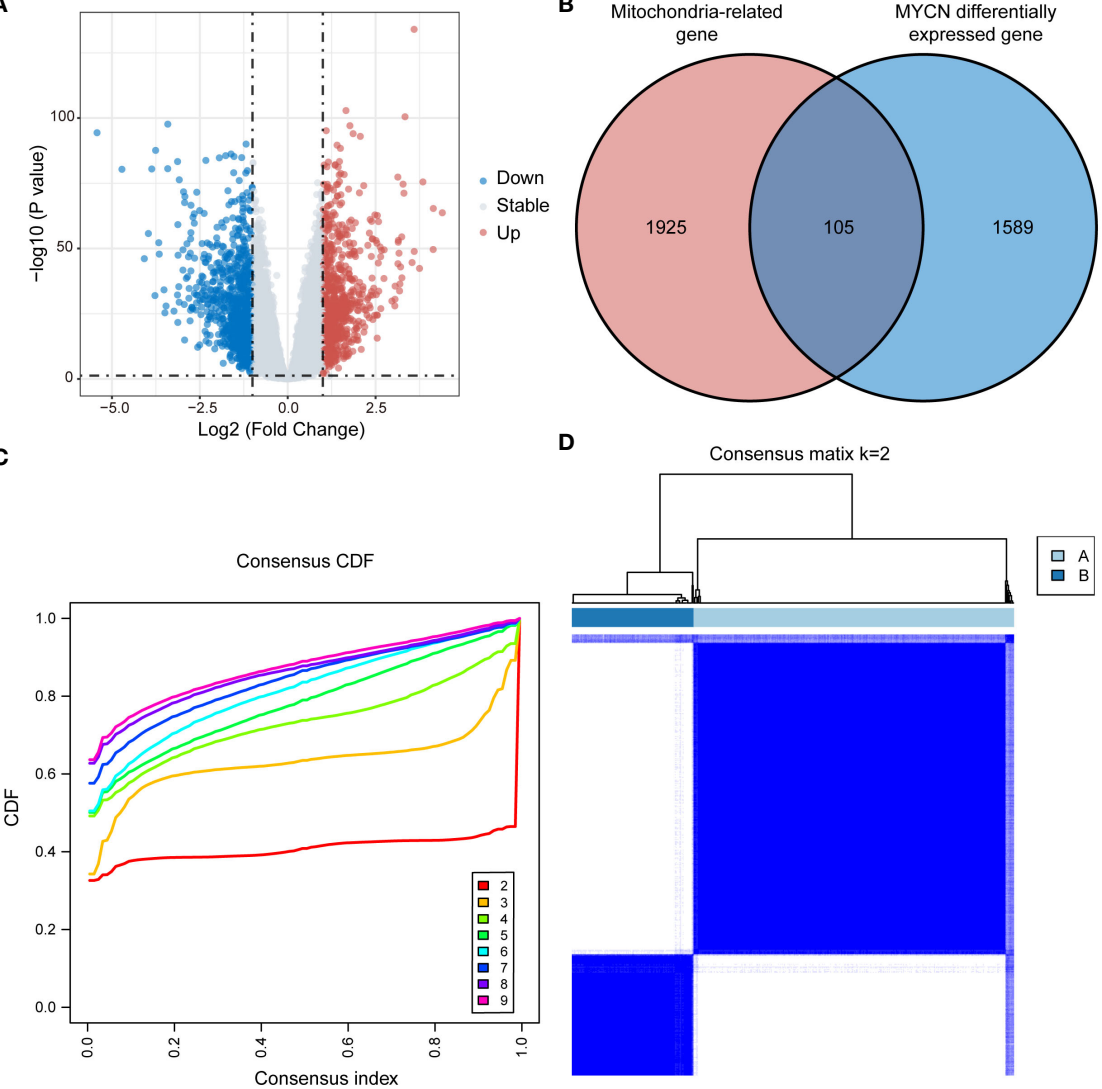
Consensus clustering in GSE49710 based on MRGs associated with NB. **(A)** Volcano plot displaying DEGs between MYCN-amplified and non-amplified NB patients in GSE49710 (Genes with P value < 0.05 and |log2FC| > 1 are highlighted). **(B)** Venn diagram illustrating the intersection of MYCN status DEGs with MRGs, identifying 105 MRGs specifically associated with neuroblastoma. **(C, D)** Consensus clustering of NB patients into clusters A and B based on the expression of 105 MRGs, with k=2 as the optimal cluster number. MRGs, mitochondria-related genes; NB, neuroblastoma; DEGs, differentially expressed genes; FC, fold change; CDF, cumulative distribution function.

In this study, we identified 1,694 DEGs between NB patients with and without MYCN amplification in GSE49710 dataset, employing a threshold of P value < 0.05 and |log2FC| > 1, with 730 up-regulated genes and 964 down-regulated genes (**Figure 1A**). Intersection of these 1,694 MYCN status DEGs with 2,030 MRGs yielded 105 MRGs specifically relevant to NB (**Figure 1B**). Unsupervised consensus clustering based on the expression profiles of 105 MRGs in the GSE49710 dataset stratified 498 NB patients. The consensus cumulative distribution function (CDF) plot suggested that the optimal k value was 2 (**Figure 1C**). Consequently, 498 NB patients were categorized into 2 clusters: Cluster A with 361 patients and Cluster B with 137 patients (**Figure 1D**).

**Figure 2:**
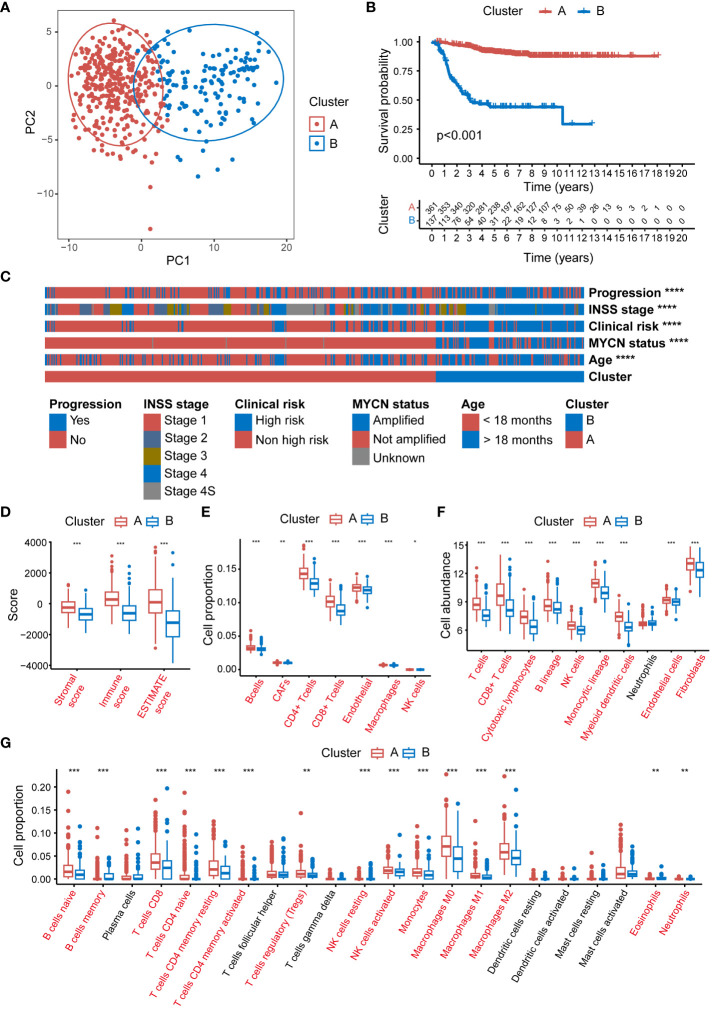
Significant disparities between Cluster A and B in GSE49710. **(A)** The PCA scatter plot demonstrating the segregation of NB patients into 2 clusters, Cluster A (blue) and B (red), based on MRG expression profiles. **(B)** The Kaplan-Meier survival curves depicting the OS probability for patients in clusters A and B. **(C)** The heatmap displaying the distribution of progression status, INSS stage, clinical risk, MYCN amplification status, and age in patients within Clusters A and B. **(D)** Box plots representing the ESTIMATE scores in Clusters A and B. **(E)** Box plots illustrating the proportion of various immune cells as analyzed by the EPIC algorithm in Clusters A and B. **(F)** Box plots depicting the cell abundance of different immune cell types as analyzed by the MCPcounter algorithm. **(G)** Box plots detailing the cell proportion of various immune cells as analyzed by the CIBERSORT algorithm in Cluster A and B. PCA, principal component analysis; NB, neuroblastoma; MRG, mitochondria-related genes; OS, overall survival; ESTIMATE, Estimation of STromal and Immune cells in MAlignant Tumour tissues using Expression data; EPIC, Estimating the Proportions of Immune and Cancer cells; MCPcounter, Microenvironment Cell Populations-counter; CIBERSORT, Cell-type Identification By Estimating Relative Subsets Of RNA Transcripts. (*P<0.05, **P<0.01, ***P<0.001, ****P<0.0001).

Significant disparities were observed between patients in Clusters A and B in terms of survival, clinical characteristics, and immune cell infiltration. Principal component analysis (PCA) distinctly separated the 2 clusters, validating the classification robustness (**Figure 2A**). The expression heatmap of the 105 MRGs in Clusters A and B was showed in **Supplementary Figure 1**. The K-M survival analysis indicated that NB patients in Cluster B had significantly worse overall survival (OS) compared to those in Cluster A (P < 0.001) (**Figure 2B**). Further analysis of clinical features showed substantial statistical differences between the 2 clusters in key clinical indicators (**Figure 2C**). Patients for progression and INSS stage 4 (an independent risk factor for NB) (49) were predominantly found in Cluster B (P < 0.0001 for both). Similarly, patients with clinical risk factors, MYCN amplification, and age below 18 months (a factor associated with poorer prognosis) were significantly concentrated in Cluster B (P < 0.0001 for all comparisons). Detailed relations between each clinical characteristic and distribution across Clusters A and B are depicted using Sankey diagrams in **Supplementary Figure 2**. Additionally, a marked difference in the TME between Clusters A and B was uncovered through 4 distinct immune infiltration analysis algorithms. The ESTIMATE algorithm suggested that patients in Cluster A had higher scores overall in terms of ESTIMATE, immune, and stromal scores compared to Cluster B (**Figure 2D**). Results from the EPIC algorithm revealed significant statistical differences in cell proportions of all 7 cell types between the two clusters (**Figure 2E**). In parallel, the analysis using MCPcounter indicated that the cell abundance of T cells, Cytotoxic lymphocytes, B lineage, NK cells, Monocytic lineage, and Myeloid dendritic cells in Cluster A was statistically higher compared to Cluster B (**Figure 2F**). Finally, the CIBERSORT analysis also reflected that Cluster A patients exhibited higher cell proportions for various immune cells compared to Cluster B (**Figure 2G**).

**Figure 3:**
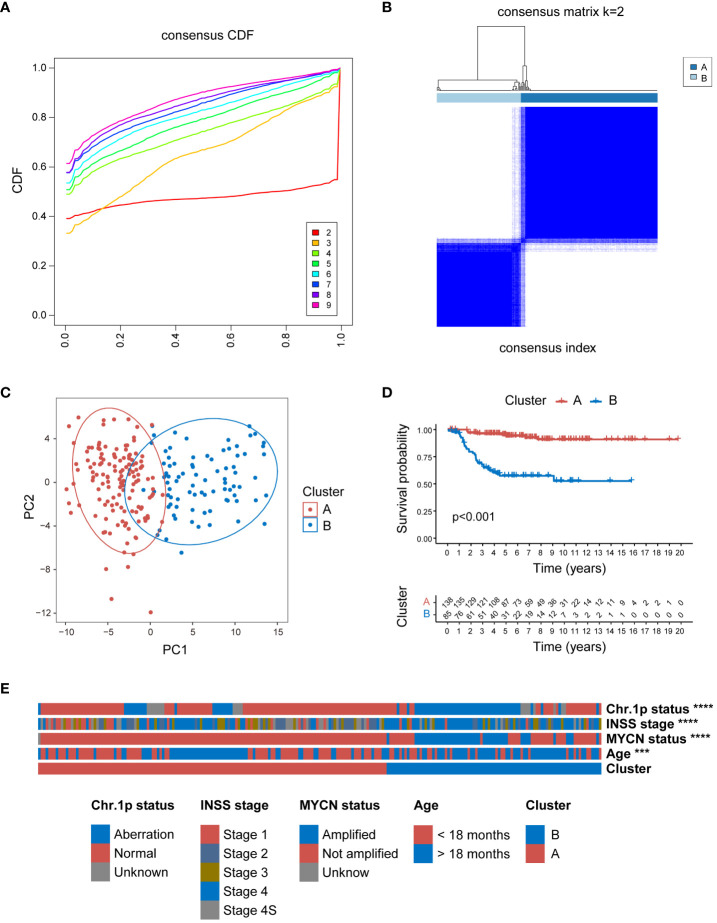
Consensus clustering of E-MTAB-8248 dataset into 2 distinct clusters. **(A)** The consensus CDF plot identifying the optimal cluster number (k=2). **(B)** Consensus matrix heatmap at k=2, displaying the robust bifurcation of the dataset into Clusters A and B. **(C)** The PCA demonstrating a clear separation between the two clusters, validating the clustering approach. **(D)** Kaplan-Meier survival analysis revealing a significant survival disadvantage for Cluster B compared to Cluster A (p<0.001). **(E)** The clinical characteristic heatmap exhibiting distinct profiles between Cluster A and B, with chromosome 1p status, INSS stage, MYCN amplification status, and age at diagnosis, underscoring the clinical relevance of the clustering. Chr: chromosome; CDF, cumulative distribution function; PCA, principal component analysis. (***P<0.001, ****P<0.0001).

To further validate the broad applicability of the clustering based on MRGs, the same cluster analysis was conducted on the E-MTAB-8248 dataset. The consensus CDF plot suggested an optimal number of clusters k = 2 (**Figure 3A**), subdividing the 223 NB children in the dataset into 138 in Cluster A and 85 in Cluster B (**Figure 3B**). The heatmap of the expression of 105 MRGs for Clusters A and B within the E-MTAB-8248 dataset is presented in **Supplementary Figure 3**. The PCA confirmed robust separation between the clusters (**Figure 3C**). Echoing the results from the GSE49710 dataset, K-M survival analysis within E-MTAB-8248 also demonstrated poorer OS for patients in Cluster B (P<0.001) (**Figure 3D**). Substantial differences were also observed between clusters A and B in important clinical characteristics within E-MTAB-8248 (**Figure 3E**). Chromosome 1p aberrations, recognized as markers of poor prognosis in NB patients (49), were more frequently observed in patients of Cluster B (P<0.0001). Similarly, patients associated with poor prognostic factors such as INSS stage 4, MYCN amplification, and age under 18 months were predominantly found in Cluster B (all comparisons P<0.0001). **Supplementary Figure 4** employs Sankey diagrams to detail the distribution of these significant clinical features between clusters A and B in the E-MTAB-8248 dataset.

### 3.2 Construction and internal validation of the MRGs-related signature

**Figure 4:**
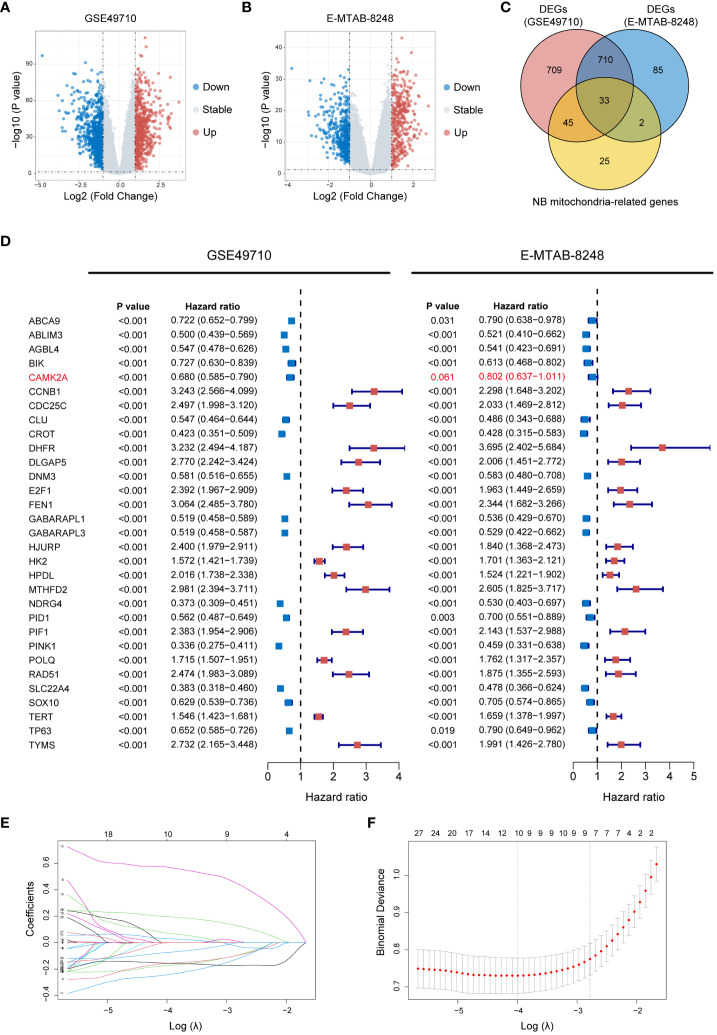
Construction of the MRGs-related signature. **(A)** Volcano plot displaying DEGs between Cluster A and B in GSE49710 (Genes with P value < 0.05 and |log2FC| > 1 are highlighted). **(B)** Volcano plot illustrating DEGs between Cluster A and B in E-MTAB-8248 (Genes with P value < 0.05 and |log2FC| > 1 are highlighted). **(C)** Venn diagram demonstrating the 33 intersecting genes found between the 1,497 DEGs in GSE49710, the 830 DEGs in E-MTAB-8248, and the 105 MRGs specific to NB. **(D)** Forest plots of HR for the 33 intersecting genes from GSE49710 and E-MTAB-8248 datasets, indicating their association with OS. P value < 0.05, and the 95% CI of HR was consistently distributed ipsilateral to 1 was considered statistically significant. **(E)** LASSO coefficient profiles of the 30 candidate genes. **(F)** Cross-validation for tuning parameter selection in the LASSO model used in **(E)**. MRGs, mitochondria-related genes; DEGs, differentially expressed genes; FC, fold change; NB, neuroblastoma; HR, hazard ratio; OS, overall survival; CI, confidence interval; LASSO, Least Absolute Shrinkage and Selection Operator.

In an effort to delineate the 2 clusters formed by MRG expression, this study constructed an MRGs-related signature to quantify the distinction through a scoring mechanism. Initially, 1,497 DEGs (618 up-regulated genes and 879 down-regulated genes) between Clusters A and B in the GSE49710 dataset (**Figure 4A**), and 830 DEGs (369 up-regulated genes and 461 down-regulated genes) between Clusters A and B in the E-MTAB-8248 dataset (**Figure 4B**) were identified (using a threshold of P < 0.05 and |log2FC| > 1). A Venn diagram depicts the 33 intersecting genes found between the 1,497 DEGs in GSE49710, the 830 DEGs in E-MTAB-8248, and the 105 MRGs specific to NB (**Figure 4C**). Further, the prognostic implications of the 33 intersecting genes were validated in both the GSE49710 and E-MTAB-8248 datasets, using K-M analysis with OS as the endpoint, categorized by the gene median expression. In GSE49710, K-M analysis revealed statistical survival differences between patients with high and low expression of each of the 33 genes (**Supplementary Figure 5**). Similarly, in the E-MTAB-8248 dataset, 31 of the 33 genes showed statistically significant survival correlations in K-M analysis (**Supplementary Figure 6**). Subsequent analysis involved univariate Cox regression to further screen for genes significantly associated with prognosis. Each of the 31 genes underwent univariate Cox regression analysis with OS as the endpoint in both GSE49710 and E-MTAB-8248 (**Figure 4D**), with only one gene (marked in red in **Figure 4D**) not showing statistical significance in E-MTAB-8248. Consequently, the remaining 30 genes proceeded to the next phase of analysis. The LASSO regression analysis was applied in GSE49710 to refine the selection to 10 genes, assigning coefficients to each (**Figures 4E, F**). The mtScore was defined as the linear combination of the mRNA expression levels of the 10 genes, weighted by their respective coefficients provided by LASSO analysis within GSE49710. The formula is as follows: mtScore = (-0.243142218 × the expression of DNM3) + (-0.173523906 × the expression of AGBL4) + (-0.156591746 × the expression of CROT) + (-0.155479922 × the expression of SLC22A4) + (-0.074329063 × the expression of TP63) + (-0.001579582 × the expression of PID1) + (0.029877545 × the expression of HK2) + (0.072465418 × the expression of DLGAP5) + (0.196643607 × the expression of TERT) + (0.570707127 × the expression of FEN1).

**Figure 5:**
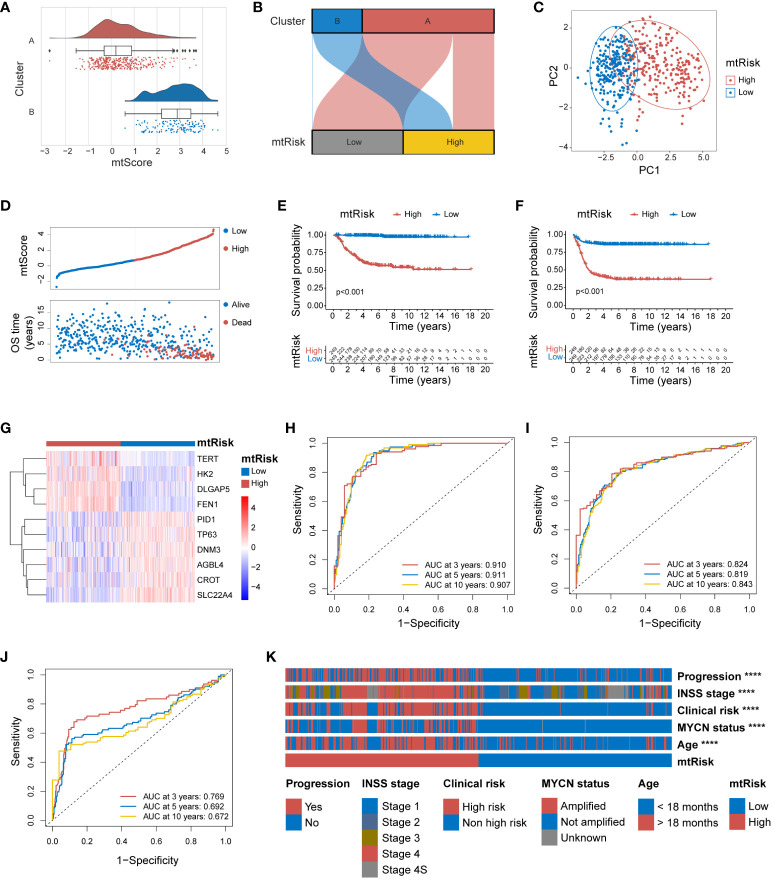
Internal validation of the MRGs-related signature in GSE49710. **(A)** Combined violin and box plot with overlaid scatter plot illustrating the distribution of mtScores across clusters A and B. **(B)** Sankey diagram demonstrating the effective discrimination of Clusters A and B using mtRisk. **(C)** PCA plot showing the clear separation between the patients of high and low mtRisk groups. **(D)** Bipartite plot of mtScore against OS time, highlighting the survival status (Alive or Dead) of patients. **(E)** Kaplan-Meier survival curve for OS, between the high and low mtRisk groups. **(F)** Kaplan-Meier survival curve for EFS, between the high and low mtRisk groups. **(G)** Heatmap showing the gene expression profiles of the 10 genes constituting the mtScore across the mtRisk stratified patient groups. **(H)** ROC curve of mtScore for OS. **(I)** ROC curve of mtScore for EFS. **(J)** ROC curve of MYCN for OS. **(K)** Heatmap displaying the association of clinical features with the mtRisk groups. MRGs, mitochondria-related genes; PCA, principal component analysis; OS, overall survival; EFS, event-free survival; ROC, receiver operating characteristic; AUC, area under the curve. (****P<0.0001).

The mtScore for all 498 patient samples in the GSE49710 dataset was calculated in this study. Following the computation of mtScores, patients were dichotomized into two risk categories, low and high mtRisk, based on the median mtScore value. Internal validation of the predictive value of mtScore and mtRisk was conducted in the GSE49710 dataset. Initially, **Figures 5A** and **B** demonstrated that mtScore and mtRisk could effectively discriminate between Clusters A and B, with Cluster B patients exhibiting higher mtScores. The PCA indicated a clear distinction between high and low mtRisk groups (**Figure 5C**). A bipartite plot of mtScore distribution revealed a concentration of dead patients within the high mtScore group (**Figure 5D**). The K-M analysis with endpoints of OS (**Figure 5E**) and Event-Free Survival (EFS) (**Figure 5F**) showed that high mtRisk NB patients faring worse in both OS and EFS compared to their low mtRisk counterparts. The heatmap in **Figure 5G** displayed the expression patterns of the 10 genes used in mtScore calculation, with TERT, HK2, DLGAP5, and FEN1 being overexpressed in high mtRisk patients, while PID1, TP63, DNM3, AGBL4, CROT, and SLC22A4 showed lower expression in the high mtRisk group. The ROC curve illustrated the prognostic prediction capability and accuracy of mtScore. The AUCs of the ROC curve for OS at 3, 5, and 10 years were 0.910, 0.911, and 0.907, respectively (**Figure 5H**), while for EFS, they were 0.824, 0.819, and 0.843 (**Figure 5I**). In contrast, the AUCs of the MYCN, a well-established biological indicator of poor prognosis in NB, prediction for OS at 3, 5, and 10 years were only 0.769, 0.692, and 0.672 (**Figure 5J**). Furthermore, the high and low mtRisk patient groups correlated well with key clinical features (**Figure 5K**), with significantly more patients with progression, INSS stage 4, clinical risk, MYCN amplification, and age under 18 months in the high mtRisk group (P < 0.0001 for all 5 comparisons). The detailed distribution of mtScores across different clinical features is presented as violin plots in **Supplementary Figure 7**.

### 3.3 External validation of the MRGs-related signature

**Figure 6:**
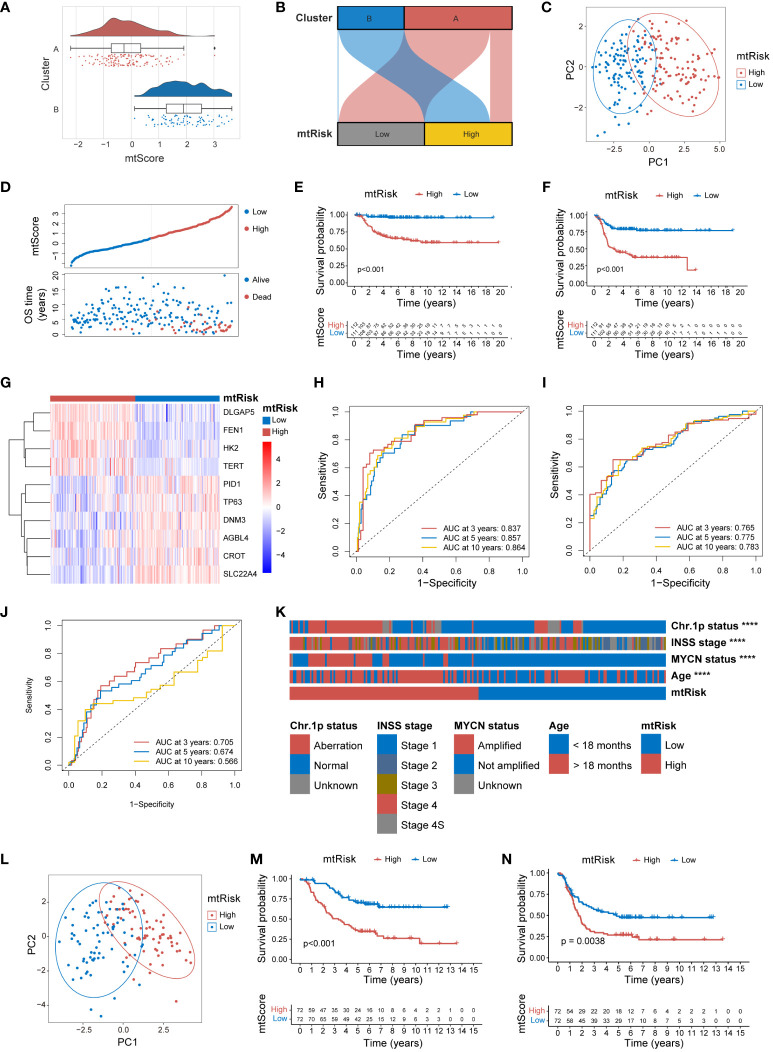
External validation of MRGs-related signature in the E-MTAB-8248 and TARGET-NBL datasets. **(A)** Combined violin, box, and scatter plot demonstrating mtScore distribution across Clusters A and B in the E-MTAB-8248 dataset. **(B)** Sankey diagram demonstrating the correlation between mtRisk stratification and Cluster A and B designation in E-MTAB-8248. **(C)** PCA plot distinctly separating high and low mtRisk patient groups in the E-MTAB-8248 dataset. **(D)** Scatter plot of mtScore against OS time in E-MTAB-8248, distinguishing patients by survival status. **(E)** Kaplan-Meier survival curve for OS in E-MTAB-8248 stratified by mtRisk. **(F)** Kaplan-Meier survival curve for EFS in E-MTAB-8248stratified by mtRisk. **(G)** Heatmap of the expression of 10 genes constituting the mtScore in E-MTAB-8248, differentiated by mtRisk groups. **(H)** ROC curve of mtScore predictive capacity for OS in E-MTAB-8248. **(I)** ROC curve of mtScore predictive capacity for EFS in E-MTAB-8248. **(J)** ROC curve of MYCN predictive capacity for OS in E-MTAB-8248. **(K)** Heatmap correlating mtRisk with key clinical features in E-MTAB-8248. **(L)** PCA plot distinctly separating high and low mtRisk patient groups in the TARGET-NBL dataset. **(M)** Kaplan-Meier survival curve for OS in TARGET-NBL categorized by mtRisk. **(N)** Kaplan-Meier survival curve for EFS in TARGET-NBL categorized by mtRisk. MRGs, mitochondria-related genes; PCA, principal component analysis; OS, overall survival; EFS, event-free survival; ROC, receiver operating characteristic; AUC, area under the curve; Chr, chromosome. (****P<0.0001).

To ascertain the general applicability of the MRGs-related signature and its quantitative indices, mtScore and mtRisk, further external validation was undertaken in the E-MTAB-8248 and TARGET-NBL datasets. Within the E-MTAB-8248 dataset, mtScore corresponded well with the identified Cluster A and B. Both the **Figures 6A** and **B** effectively demonstrated that mtScore and mtRisk could distinguish between Clusters A and B, with Cluster B associated with higher mtScores and mtRisk. the PCA underscored that patients in the E-MTAB-8248 dataset could be well-separated into high and low mtRisk groups (**Figure 6C**). The bipartite distribution plot revealed that dead patients predominantly occupied the high mtScore sector (**Figure 6D**). K-M analysis for OS (**Figure 6E**) and EFS (**Figure 6F**) were performed in E-MTAB-8248, demonstrating that high mtRisk patients had poorer prognoses compared to their low mtRisk counterparts (P < 0.01 for both). The heatmap in **Figure 6G** illustrates the expression of the 10 genes comprising the mtScore in high and low mtRisk patients within the E-MTAB-8248 dataset. The ROC curves displayed the predictive capacity of mtScore, with AUC values for OS at 3 years (0.837), 5 years (0.857), and 10 years (0.864) (**Figure 6H**), and for EFS at 3 years (0.765), 5 years (0.775), and 10 years (0.783) (**Figure 6I**). In comparison, the AUCs of the MYCN prediction for OS were only 0.705, 0.674, and 0.566 at the same time points (**Figure 6J**). The mtRisk also showed significant statistical correlation with key clinical features in the E-MTAB-8248 dataset (**Figure 6K**), where more malign clinical features such as chromosome 1p aberration, INSS stage 4, MYCN amplification, and age <18 months were significantly more prevalent in the high mtRisk group (P<0.0001 for all comparisons). The distribution of mtScores across different clinical feature groups is presented as violin plots in **Supplementary Figure 8**.

External validation of mtScore and mtRisk in the TARGET-NBL dataset reiterated the robust predictive power of the MRGs-related signature. PCA delineated a clear distinction between high and low mtRisk groups in the TARGET-NBL dataset (**Figure 6L**). K-M survival analysis confirmed the consistent predictive power of mtRisk for prognosis, with high mtRisk patients exhibiting worse outcomes in both OS (**Figure 6M**) and EFS (**Figure 6N**) endpoints (P<0.001 and P<0.01, respectively). Beyond prognosis, mtRisk was also correlated with important clinical features in the TARGET-NBL dataset (**Supplementary Figure 9A**). High-risk COG risk group, unfavorable histology, high MKI, MYCN amplification, INSS stage 4, and age <18 months were statistically more frequent in high mtRisk patients. **Supplementary Figures 9B–G** display the mtScore distributions for different clinical feature groups as violin plots.

### 3.4 Predictive efficacy of MRGs-related signature for TIME, stemness, and chemosensitivity

**Figure 7:**
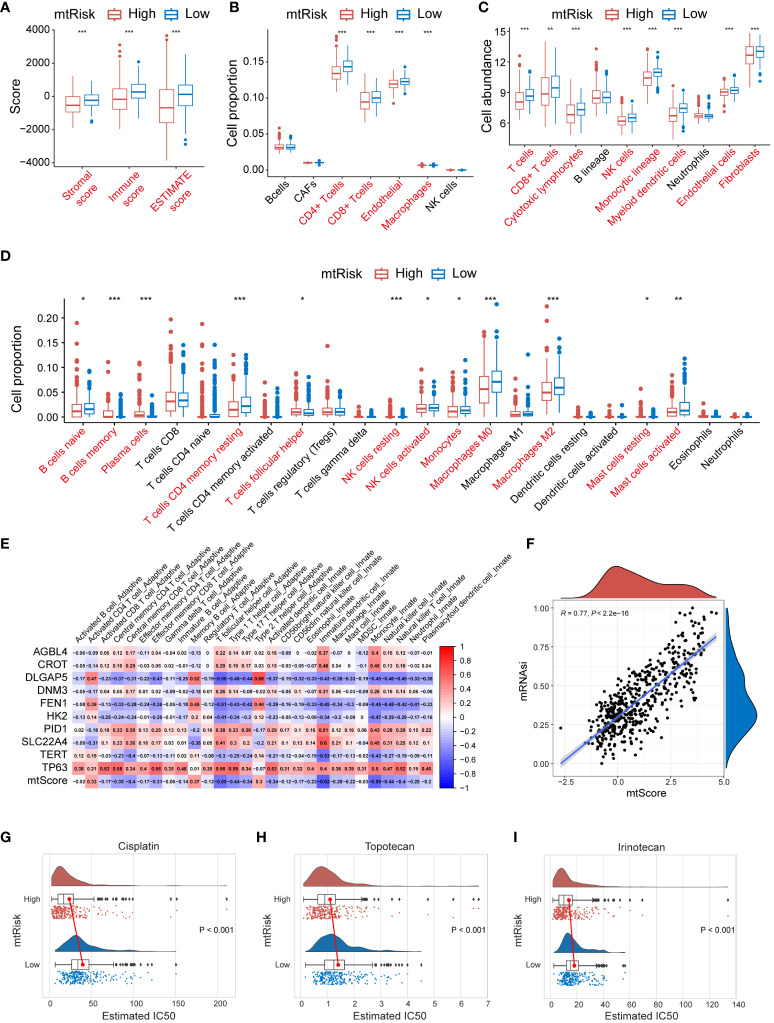
Immune profile, stemness, and drug Sensitivity analysis related to MRGs-related signature in GSE49710. **(A)** Box plots representing the ESTIMATE scores stratified by mtRisk groups. **(B)** Box plots illustrating the proportion of various immune cells as analyzed by the EPIC algorithm, stratified by mtRisk groups. **(C)** Box plots depicting the cell abundance of different immune cell types as analyzed by the MCPcounter algorithm. **(D)** Box plots detailing the cell proportion of various immune cells as analyzed by the CIBERSORT algorithm stratified by mtRisk groups. **(E)** Heatmap displaying the correlation between the mtScore, the ten genes comprising mtScore, and 28 immune cell types. **(F)** Scatter plot demonstrating a positive association between mtScore and mRNAsi. **(G–I)** Violin plots illustrating the estimated IC50 values for Cisplatin **(G)**, Topotecan **(H)**, and Irinotecan **(I)**, comparing high and low mtRisk groups. MRGs, mitochondria-related genes; ESTIMATE, Estimation of STromal and Immune cells in MAlignant Tumour tissues using Expression data; EPIC, Estimating the Proportions of Immune and Cancer cells; MCPcounter, Microenvironment Cell Populations-counter; CIBERSORT, Cell-type Identification By Estimating Relative Subsets Of RNA Transcripts; IC50, half-maximal inhibitory concentration. (*P<0.05, **P<0.01, ***P<0.001).

The MRGs-related signature and its quantitative markers, mtScore and mtRisk, developed in this study, not only predict the prognosis of NB patients but also show significant relevance to immune infiltration in the TME. Various algorithms were applied to assess the indication of mtScore and mtRisk towards immune infiltration within the GSE49710 dataset. The ESTIMATE algorithm indicated that patients with low mtRisk had higher scores, with higher total ESTIMATE score, immune score, and stromal score compared to patients with high mtRisk (**Figure 7A**). According to the EPIC algorithm, the proportion of immune cells such as CD4^+^ T cells, CD8^+^ T cells, and macrophages was statistically higher in the TIME of patients with low mtRisk compared to those with high mtRisk (**Figure 7B**). The MCPcounter algorithm suggested that the cell abundance of T cells, CD8^+^ T cells, cytotoxic lymphocytes, NK cells, monocytic lineage, myeloid dendritic cells, endothelial cells, and fibroblasts was higher in the low mtRisk patient group than in the high mtRisk group (**Figure 7C**). CIBERSORT analysis revealed statistically significant differences in the cell proportions of naive B cells, memory B cells, plasma cells, resting memory CD4^+^ T cells, follicular helper T cells, resting NK cells, activated NK cells, monocytes, M0 macrophages, M2 macrophages, resting mast cells, and activated mast cells between the high and low mtRisk groups (**Figure 7D**). Additionally, a heatmap displayed the correlation between mtScore, the 10 genes constituting mtScore, and 28 types of immune cells (**Figure 7E**).

Furthermore, results demonstrated that mtScore is significantly positively correlated with tumor cell stemness. A scatter plot in **Figure 7F** exhibited the relationship between mtScore and mRNAsi values in the GSE49710 dataset. The scatter plot revealed a strong positive correlation, with an R value of 0.77 (P < 0.0001). A trend line drawn through the data points further emphasized this positive linear relationship.

Moreover, mtRisk may also predict the sensitivity to 3 commonly used drugs in NB patients. Results showed that patients in the low mtRisk group had statistically significantly higher IC50 values for Cisplatin (**Figure 7G**), Topotecan (**Figure 7H**), and Irinotecan (**Figure 7I**) compared to the high mtRisk group, suggesting increased sensitivity to these 3 drugs in the high mtRisk patients (P < 0.001 for 3 comparison).

### 3.5 Analysis of FEN1’s essential role through single-cell transcriptome sequencing data

**Figure 8:**
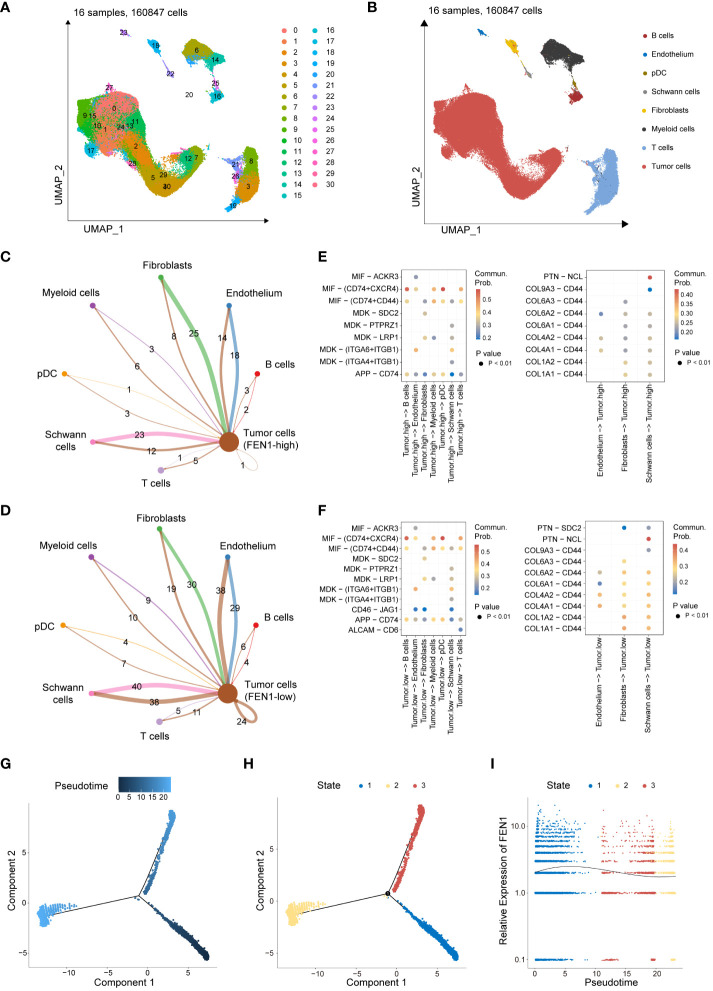
Single-cell transcriptomic data analysis revealing the role of FEN1 in NB tumor cells. **(A)** UMAP dimensionality reduction and clustering of 160,847 cells from 16 neuroblastoma patient samples. **(B)** Annotated UMAP Clustering of Cell Types. **(C)** Cell-cell communication counts network of FEN1-high tumor cells with the surrounding microenvironment. **(D)** Cell-cell communication counts network of FEN1-low tumor cells with the surrounding microenvironment. **(E)** Heatmap illustrating significant ligand-receptor pairs between FEN1-high tumor cells and other cell types. **(F)** Heatmap illustrating significant ligand-receptor pairs between FEN1-low tumor cells and other cell types. **(G)** Pseudotime trajectory analysis of NB tumor cells, with color gradient indicating progression through pseudotime. **(H)** Cells are categorized into 3 developmental states along the pseudotime trajectory. **(I)** Trend of FEN1 gene expression across pseudotime, demonstrating its dynamic changes during tumor cell development. NB, neuroblastoma; UMAP, uniform manifold approximation and projection; pDC, plasmacytoid dendritic cell; Commun Prob, communication probability.

This study further validated the significant function and role of FEN1 in NB, the gene with the highest contribution in the MRGs-related signature. Quality control, normalization, and batch effect removal were initially applied to single-cell transcriptomic sequencing data from 160,847 cells of 16 NB patients sourced from GSE137804. Subsequent dimensionality reduction via UMAP clustered the cells into 30 clusters (**Figure 8A**), and cell type annotation using known markers identified 8 cell types similar to the original GSE137804 study (22), including tumor cells, T cells, B cells, endothelium, plasmacytoid dendritic cell (pDC), Schwann cells, fibroblasts, and myeloid cells (**Figure 8B**). The markers used for cell annotation are presented in **Supplementary Figure 10A**.

Tumor cells were segregated into FEN1-high and FEN1-low groups based on the median expression of FEN1. Cell-cell communication analysis further investigated differences between FEN1-high and FEN1-low tumor cells in their interactions with the TME. Compared to FEN1-high tumor cells, FEN1-low tumor cells exhibited increased and stronger communication with surrounding cells. **Figures 8C, D** respectively show the communication counts of tumor cells (FEN1-high) and tumor cells (FEN1-low) with adjacent cells. **Supplementary Figures 10B, C** display the communication weights of tumor cells (FEN1-high) and tumor cells (FEN1-low), respectively. Additionally, the exploration of interacting pairs in cell communication revealed that tumor cells primarily interact with other cells via the MIF-(CD74+CXCR4) axis; conversely, other cells predominantly communicate with tumor cells through the PTN-NCL axis (**Figures 8E, F**).

Pseudotime trajectory analysis illustrated the temporal expression changes of FEN1 in the development of NB tumor cells. **Figure 8G** depicts the pseudotime trajectory of NB tumor cells, with the cell developmental trajectory divided into 3 states in **Figure 8H**. The gene expression trend of FEN1 over pseudotime is shown in **Figure 8I**, indicating that FEN1 expression is higher in the early stages of NB tumor cell development than in later stages.

### 3.6 Functional validation of FEN1 in NB cell lines

**Figure 9:**
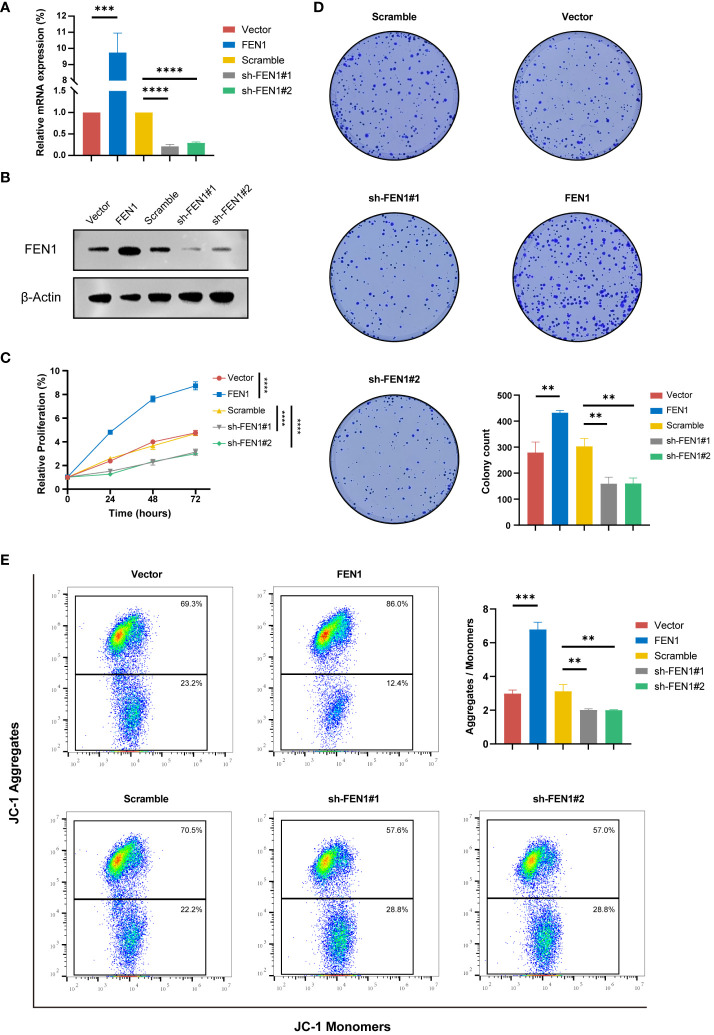
Functional validation of FEN1 OE and KD in NB cell lines. **(A)** qRT-PCR Validation of FEN1 OE and KD Efficiency at RNA Level in NB Cells. **(B)** Western Blot Confirmation of FEN1 OE and KD at Protein Level in NB Cells. **(C)** Line graph depicting the relative proliferation of NB cells, measured by CCK-8 assay. **(D)** Representative images and quantification of colony formation assay results. **(E)** Flow cytometry analysis of JC-1 staining to assess MMP (ΔΨm), with the Aggregates/Monomers ratio indicating changes of apoptosis. OE, overexpression; KD, knockdown; NB, neuroblastoma; MMP, mitochondrial membrane potential. (**P<0.01, ***P<0.001, ****P<0.0001).

The current study further investigated the important role of FEN1 in NB cell by modulating its expression through lentiviral-mediated OE or KD in human NB cells. Successful modulation of FEN1 at both RNA and protein levels was confirmed by qRT-PCR (**Figure 9A**) and Western blot (**Figure 9B**), achieving the desired OE and KD effects.

The impact of FEN1 on NB cell proliferation was assessed using the CCK-8 assay (**Figure 9C**). Cells with FEN1 OE showed significantly higher proliferation rates compared to the vector group (P<0.0001). Cells with FEN1 KD (sh-FEN1#1 and sh-FEN1#2), demonstrated significantly reduced proliferation compared to the scramble group (P<0.0001 for both comparisons). Plate clonogenic assays corroborated these findings (**Figure 9D**), where FEN1 OE increased colony formation in NB cells compared to NC (P<0.01), and KD led to a significant decrease in colony numbers (P<0.01 for both sh#1 and sh#2).

To explore changes in apoptosis following FEN1 modulation, we quantified ΔΨm using the JC-1 dye. An increase in red/green fluorescence ratio, indicating higher J-aggregates formation, reflects higher MMP and thus, a lower level of apoptosis. **Figure 9E** shows that cells with FEN1 OE had a significantly higher Aggregates/Monomer ratio compared to NC (P<0.001), whereas FEN1 KD cells displayed a significant reduction, indicating increased apoptosis (P<0.01 for both sh#1 and sh#2).

**Figure 10:**
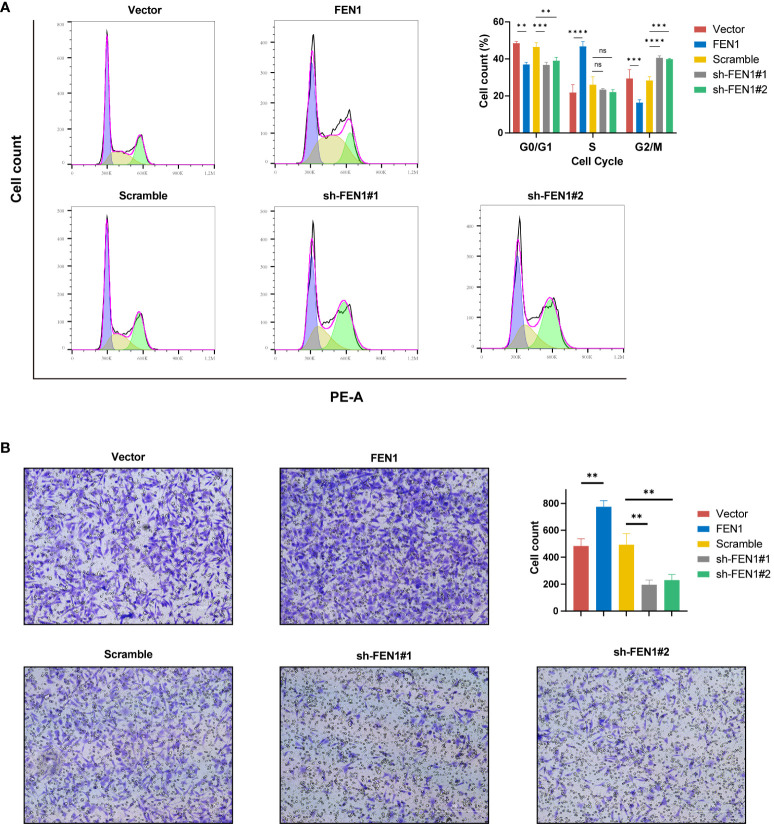
Impact of FEN1 modulation on NB cell cycle progression and invasiveness. **(A)** Flow cytometry cell cycle analysis presenting the situation of NB cells in 5 group. **(B)** Transwell invasion assays with representative images and quantitative analysis displaying the invasive capacity of NB cells in 5 groups. NB, neuroblastoma; ns, not significant. (**P<0.01, ***P<0.001, ****P<0.0001).

Cell cycle analysis also yielded positive findings (**Figure 10A**), with FEN1 KD cells showing an increased proportion in the G2/M phase, suggesting G2/M arrest compared to the NC (P<0.0001 for sh#1, P<0.001 for sh#2). Cells OE FEN1 exhibited a significant increase in the S phase proportion compared to NC (P<0.0001), indicative of heightened DNA synthesis and replication activity.

Furthermore, FEN1 may influenced the invasive capacity of NB cells. Transwell assays revealed changes in NB cell invasion following FEN1 OE and KD (**Figure 10B**). Compared to NC, FEN1 OE enhanced the invasiveness of NB cells, with increased cell numbers traversing the membrane (P<0.01). Conversely, FEN1 KD reduced NB cell invasion, with fewer cells penetrating the membrane (P<0.01 for both sh#1 and sh#2).

## 4 Discussion

Our study has successfully constructed a prognostic signature based on MRGs and developed quantitative indices, namely mtScore and mtRisk. Through extensive internal and external validation, the MRGs-related signature exhibited superior prognostic predictive effect and value over the traditional molecular marker MYCN in NB patients. Furthermore, it demonstrated predictive capability for immune infiltration in the TME, tumor cell stemness, and sensitivity to specific chemotherapeutic agents. Furthermore, through single-cell transcriptomic analysis, we underscored the pivotal role played by FEN1, the most contributive molecule in the MRGs-related signature, in the molecular crosstalk and developmental trajectory of NB cells. The experimental validation results underscored that FEN1 expression significantly affects processes such as cell proliferation, apoptosis, cell cycle progression, and invasiveness in NB cells.

In this study, MRGs effectively stratified NB patients into 2 distinct groups with significant differences in prognosis and immune infiltration characteristics. An MRGs-related signature and its quantitative metrics, mtScore and mtRisk, were developed to characterize these 2 patient groups, revealing that lower mtScore and mtRisk are associated with significantly better prognoses. Similar works have been reported in colorectal cancer, hepatocellular carcinoma, and other cancers (50, 51), but this is the first time an MRGs-related signature has been identified and explored in NB, to our knowledge. It’s noteworthy that the predictive efficiency of this MRGs-related signature for NB patients surpasses that of MYCN, traditionally recognized as the best genetic marker for forecasting outcomes in NB (52, 53). This highlights the potential of MRGs-related signature to provide a more comprehensive understanding of NB prognosis beyond conventional markers.

In line with the above results, MRGs-related signature not only predicts prognosis, but also identifies key clinical features to a certain extent. MYCN amplification, which is closely related to the poor prognosis of high-risk NB patients (54), was found to be significantly correlated with high mtRisk in this study. INSS4 stage, as an independent risk factor for NB (49), was found to be significantly associated with high mtRisk in this study. Similarly, high mtRisk was profoundly associated with a spectrum of adverse clinical features, including clinical risk, progression, chromosomal 1p aberration, high MKI, unfavorable histology, and high COG risk. The congruence of the MRGs-related signature’s predictive capacity for both prognosis and key clinical features in NB patients underscores its utility beyond mere prognostic estimation.

The Further analysis of immune infiltration implies that patients with lower mtScore and mtRisk tend to have a more active immune environment within their tumors. This heightened immune activity is hypothesized to be a key factor contributing to their better prognosis, suggesting a link between mitochondrial function, immune engagement, and cancer outcome. Exploring the TIME and transitioning from “cold” to “hot” tumors could significantly enhance therapeutic efficacy in NB, a tumor traditionally marked by immune suppression and modest responses to immunotherapy (5). The establishment of the MRGs-related signature provides a promising avenue for identifying and modulating the TME. By targeting specific MRGs to activate the immune landscape within NB, it may be possible to convert these traditionally “cold” tumors into “hot” tumors, potentially making them more amenable to immunotherapeutic interventions. This approach, aligning with findings in other cancers, underscores the critical interplay between mitochondrial dynamics and immune responsiveness in determining cancer prognosis and treatment outcomes (14, 55). The results of this study may provide a new target for immunotherapy of NB and other tumors (56–59).

In addition, the significant linear correlation between mtScore and mRNAsi underscores the potential of our developed MRGs-related signature to effectively indicate the stemness of tumor cells in patients. Our team’s previous work has developed an mRNAsi-based risk score for NB, which demonstrated excellent performance in predicting patient prognosis, immune infiltration, and treatment response (60). This study extends the prognostic utility of mtScore as a marker of stemness, potentially impacting the clinical management of NB significantly. Patients with elevated mtScores might be identified as harboring a higher burden of tumor stem cells, likely to undergo aggressive disease progression and exhibit poor responses to standard therapies. This observation aligns with the outcomes of poor prognosis and reduced sensitivity to certain chemotherapies among patients with high mtScores. Such insights potentially could facilitate the stratification of patients into more personalized treatment regimens.

In the realm of clinical pharmacotherapy, our findings highlight a significant role of mtRisk in shaping therapeutic responses. At first glance, patients categorized under high mtRisk appear to present a formidable challenge in treatment management due to their poor prognosis, immune-suppressive TME, and pronounced tumor cell stemness. However, a pivotal discovery of our study is the heightened sensitivity of high mtRisk patients to 3 clinically prevalent drugs for NB: Cisplatin, Topotecan, and Irinotecan. All 3 drugs are internationally recognized for the treatment of NB patients (61–63). This enhanced drug responsiveness, surpassing that of low mtRisk patients, uncovers a nuanced aspect of mtRisk’s clinical implications. This holds some promise for the therapeutic management of patients with high mtRisk, and also re-emphasizes the importance of precise individualized treatment in highly heterogeneous NB patients.

The MRGs-related signature in NB is composed of ten genes: FEN1, TERT, DLGAP5, HK2, PID1, TP63, SLC22A4, CROT, AGBL4, DNM3. FEN1 (Flap endonuclease 1) is crucial in DNA replication and repair, and its overexpression is linked to poor prognosis in various cancers, indicating its potential as a target for cancer therapy (64). TERT (Telomerase reverse transcriptase), the catalytic subunit of telomerase, affects telomere length by affecting telomerase activity, and considered to be a useful marker in diagnosis and prognosis of various cancers and a new therapy approach (65). DLGAP5 (Discs large homolog associated protein 5) is involved in mitotic spindle assembly, and its overexpression is associated with tumor progression and adverse outcomes in cancer patients, highlighting its role in cell division and potential as a therapeutic target (66, 67). HK2 (Hexokinase 2) catalyzes the first step of glycolysis and its upregulation in tumors is linked to enhanced glycolytic metabolism typical of cancer cells, suggesting its involvement in the Warburg effect and as a target for metabolic therapy (68). The role of PID1 (Phosphotyrosine interaction domain-containing protein 1) in cancer involves modulating lipid metabolism and mitochondrial function, indicating its potential impact on tumor metabolic reprogramming and its association with cancer progression (69, 70). A member of the p53 family, TP63 is implicated in the development and progression of several cancers, where it can influence cell cycle regulation, apoptosis, and the immune response in the TME (71, 72). SLC22A4, a solute carrier protein, has been associated with drug disposition and response in cancer therapy, reflecting its role in modulating chemotherapeutic efficacy and resistance mechanisms in tumors (73). CROT is involved in fatty acid metabolism, and alterations in its expression are linked to changes in cancer cell metabolism and potential effects on tumor growth and patient prognosis (74). AGBL4 (ATP/GTP binding protein-like 4), as a neuronal differentiation marker, participates in neuronal differentiation by promoting mitochondrial axonal growth and axonal transport (75). DNM3 (Dynamin 3), involved in exosomes, endocytosis, and tumor metastasis, is considered as a tumor suppressor gene in a variety of cancers such as non-small-cell lung cancer (NSCLC), hepatocellular carcinoma, papillary thyroid carcinoma, and colon cancer (76–79). Therefore, the MRGs-related signature developed in this study encompasses a range of factors related to patient prognosis, the TIME, apoptosis, cell cycle progression, mitochondrial function, neuronal differentiation, and resistance mechanisms in NB. This comprehensive signature holds potential for guiding therapeutic strategies and prognosis assessment in NB patients.

The single-cell transcriptomics data analysis further explored the role of the most important gene in the MRGs-related signature, FEN1, providing profound insights into this gene’s multifaceted functions in NB. The enhanced communication between FEN1-low tumor cells and the surrounding cells, as revealed through our analysis, suggests a more dynamic interplay within the TME. Taken together with the results of bulk sequencing data analysis (FEN1 is an oncogene associated with poor prognosis in NB patients and positively contributes to mtScore), we may be able to make an important hypothesis. High FEN1 expression could lead to a reduced need for external support from the TME, suggesting that these cells might have developed autonomous signaling pathways that promote proliferation, resist apoptosis, and enhance invasion capabilities without the extensive need for stromal or immune cell interactions. This autonomy could be a factor in their increased progression and aggressiveness. The differential communication patterns, particularly through the MIF-(CD74+CXCR4) and PTN-NCL axes, may underscore specific pathways amenable to therapeutic intervention. Furthermore, pseudotime trajectory analysis elucidating the temporal changes in FEN1 expression across the developmental trajectory of NB tumor cells underscores its significance. The observation that FEN1 expression is higher in the early stages of tumor cell development suggests a role in the initial phases of tumorigenesis or in maintaining a stem-like state of the tumor cells. This finding is supported by research from Z. Peng, et al., which confirmed FEN1’s capability to promote stemness in tumor cells (80).

FEN1 is an enzyme characterized by its multifunctional enzymatic activities, playing a pivotal role in the processes of DNA replication and repair (81). The exonuclease activity of FEN1 is critical for the maturation of lagging strand Okazaki fragments during DNA replication. This activity facilitates the removal of RNA primers at the termini of these fragments, as well as the trimming of damaged ends during DNA repair mechanisms (82, 83). As an endonuclease, FEN1 recognizes and cleaves flap structures, which are intermediates formed during DNA replication. The precise excision of these flap structures by FEN1 ensures the continuity and fidelity of DNA synthesis. This endonuclease activity is particularly crucial for genomic stability, as it aids in the accurate resolution of structural anomalies encountered during DNA replication and repair processes (84). FEN1 has been implicated in the malignancy progression of various cancers, including gastric cancer, NSCLC, and cholangiocarcinoma (85–87). This study extends the understanding of FEN1’s oncogenic role by exploring its functional significance in NB cell lines through OE and KD experiments. Our findings illustrate the multifaceted role of FEN1 in modulating NB cell behaviors, emphasizing its critical involvement in cell proliferation, apoptosis, cell cycle progression, and invasiveness. The pivotal role of FEN1 in supporting NB cell proliferation and colony formation underscores its important contribution to the proliferation and growth of NB. This aligns with FEN1’s established roles in DNA replication and repair mechanisms (88). Furthermore, the regulation of FEN1 significantly impacts NB cell apoptosis and cell cycle dynamics. Increased apoptosis in FEN1 KD cells highlights a potential vulnerability that could be therapeutically exploited to induce cell death in NB cells. This observation aligns with existing research indicating FEN1’s capacity to inhibit tumor cell apoptosis (89). Cell cycle analysis revealed G2/M phase arrest in FEN1 KD cells and an increase in the proportion of cells in the S phase following FEN1 OE, further evidencing FEN1’s regulatory role in cell cycle progression. Such findings suggest that FEN1 is not only indispensable for the replication process but also crucial for the proper progression of the cell cycle, likely by ensuring the fidelity of DNA replication and repair prior to mitotic entry. Alterations in the invasive capacity of NB cells post-FEN1 modulation underscore the gene’s role in tumor metastasis. Enhanced invasiveness in FEN1 OE cells could reflect an increased ability to facilitate tumor spread. Coupled with resistance to apoptosis and increased proliferation, these capabilities position FEN1 as a key driver of NB aggressiveness and metastatic potential. In summary, our findings advocate for FEN1’s important role in NB cell proliferation, survival, and invasiveness, and emphasize that FEN1 may serve as a potential therapeutic target for NB.

This study presents a pioneering effort in constructing an MRGs-related signature for NB, revealing potential avenues for further research and clinical application. However, there are limitations to consider. Of the ten genes comprising the constructed MRGs-related signature, only one was confirmed in subsequent single-cell data analysis and experimental validation. It is imperative to highlight that the validated gene, FEN1, is not only a part of the signature but its most crucial and significantly contributory element, underscoring its pivotal role within the MRGs-related signature for NB. The roles of the remaining nine genes in NB require further exploration through more extensive data analysis and experimental investigation. Additionally, the validation of the signature and its associated genes would benefit from a broader array of NB cell lines, animal models, and clinical patient samples to confirm their function and impact more definitively. This approach underscores the importance of comprehensive validation to strengthen the findings and potential clinical applications of genomic signatures in cancer research.

In conclusion, this study shows for the first time that MRGs can divide NB patients into two clusters that differ significantly in terms of survival prognosis, clinical features, and TIME. On this basis, this study developed MRGs-related signature and its quantitative indicators mtScore and mtRisk to characterize the above two clusters. The MRGs-related signature constructed in this study can successfully distinguish heterogeneous NB patients in different clusters, which is of great significance for the targeted and precise treatment of NB patients with different characteristics. Notably, the MRGs-related signature can predict the prognosis of NB patients, and the predictive performance is better than that of MYCN. The MRGs-related signature is significantly associated with malignant clinical features including MYCN amplification status. Besides, the MRGs-related signature may indicate the immune infiltration in TIME of NB patients to a certain extent, which may be of great significance for distinguishing “hot” tumors from “cold” tumors and predicting the response of immunotherapy. The MRGs-related signature was also adept at representing tumor cell stemness, and sensitivity to the chemotherapeutic agents Cisplatin, Topotecan, and Irinotecan. Furthermore, the important role of FEN1, the most important gene in MRGs-related signature, in NB was demonstrated by single-cell data analysis and experimental validation in this study. The development of mtScore and mtRisk provides a new perspective and evidence for the precise treatment, prognosis prediction, the conversion of “cold” and “hot” tumors, and the activation of TIME of NB patients. The important role of FEN1 demonstrated in this study also provides a potential new target for the treatment of NB.

## Data availability statement

The original contributions presented in the study are included in the article/**Supplementary Material**. Further inquiries can be directed to the corresponding authors.

## Ethics statement

Ethical approval was not required for the studies on humans in accordance with the local legislation and institutional requirements because only commercially available established cell lines were used.

## Author contributions

CW: Writing – review & editing, Writing – original draft, Visualization, Validation, Software, Methodology, Investigation, Formal analysis, Data curation. JT: Writing – review & editing, Writing – original draft, Visualization, Validation, Software, Methodology, Investigation, Formal analysis, Data curation. YJ: Writing – review & editing, Writing – original draft, Visualization, Validation, Software, Methodology, Investigation, Formal analysis, Data curation. ZL: Writing – review & editing, Investigation, Data curation. JY: Writing – review & editing, Visualization, Methodology. YBJ: Writing – review & editing, Visualization, Methodology. YX: Writing – review & editing, Investigation, Data curation. BG: Writing – review & editing, Supervision, Resources, Funding acquisition. QD: Writing – review & editing, Supervision, Resources, Funding acquisition. QZ: Writing – review & editing, Supervision, Resources, Funding acquisition.
