# Supplementary material for: A mitochondria-related genes associated neuroblastoma signature - based on bulk and single-cell transcriptome sequencing data analysis, and experimental validation
Source: Front Immunol. 2024 Jun 19;15:1415736. doi: 10.3389/fimmu.2024.1415736 (PMC11220120; doi:10.3389/fimmu.2024.1415736)
Supplement: Supplementary file 1 [file DataSheet_1.pdf]

Supplementary Table 1. Baseline clinical characteristics of the datasets used in this study

	GSE49710 (N=498)	E-MTAB-8248 (N=223)	TARGET-NBL (N=144)	GSE137804 (N=16)
**Age**				
<18 months	305 (61.2%)	104 (46.6%)	27 (18.8%)	7 (43.8%)
>18 months	193 (38.8%)	119 (53.4%)	117 (81.3%)	9 (56.3%)
**MYCN status**				
Amplified	92 (18.5%)	46 (20.6%)	30 (20.8%)	3 (18.8%)
Not amplified	401 (80.5%)	176 (78.9%)	113 (78.5%)	13 (81.3%)
Unknown	5 (1.0%)	1 (0.4%)	1 (0.7%)	0 (0.0%)
**INSS stage**				
Stage 1	121 (24.3%)	29 (13.0%)	0 (0.0%)	3 (18.8%)
Stage 2	78 (15.7%)	39 (17.5%)	1 (0.7%)	0 (0.0%)
Stage 3	63 (12.7%)	36 (16.1%)	6 (4.2%)	4 (25.0%)
Stage 4	183 (36.7%)	89 (39.9%)	117 (81.3%)	8 (50.0%)
Stage 4S	53 (10.6%)	30 (13.5%)	20 (13.9%)	1 (6.3%)
**Clinical risk**				
High risk	176 (35.3%)	NA	NA	NA
Non high risk	322 (64.7%)	NA	NA	NA
**Progression**				
Yes	183 (36.7%)	NA	NA	NA
No	315 (63.3%)	NA	NA	NA
**Chr.1p status**				
Aberration	NA	67 (30.0%)	NA	NA
Normal	NA	137 (61.4%)	NA	NA
Unknown	NA	19 (8.5%)	NA	NA
**MKI**				
High	NA	NA	32 (22.2%)	NA
Intermediate	NA	NA	38 (26.4%)	NA
Low	NA	NA	45 (31.3%)	NA
Unknown	NA	NA	29 (20.1%)	NA
**Histology**				
Favorable	NA	NA	26 (18.1%)	7 (43.8%)
Unfavorable	NA	NA	108 (75.0%)	9 (56.3%)
Unknown	NA	NA	10 (6.9%)	0 (0.0%)
**COG risk group**				
High risk	NA	NA	118 (81.9%)	11 (68.8%)
Intermediate risk	NA	NA	12 (8.3%)	2 (12.5%)
Low risk	NA	NA	14 (9.7%)	3 (18.8%)
**Distant metastasis**				
Yes	NA	NA	NA	9 (56.3%)
No	NA	NA	NA	7 (43.8%)

INSS: International neuroblastoma staging system; MKI: Mitosis karyorrhexis index; COG: Children’s oncology group; NA: Not available.

Supplementary Table 2. The shRNA targeting sequences for human FEN1 gene

Gene	Sequences (5'-3')
sh-FEN1#1	TTGCCGTCTTGTACCCTTAAG
sh-FEN1#2	GCAGTGACTACTGTGAGAGTA

shRNA: short hairpin RNA.

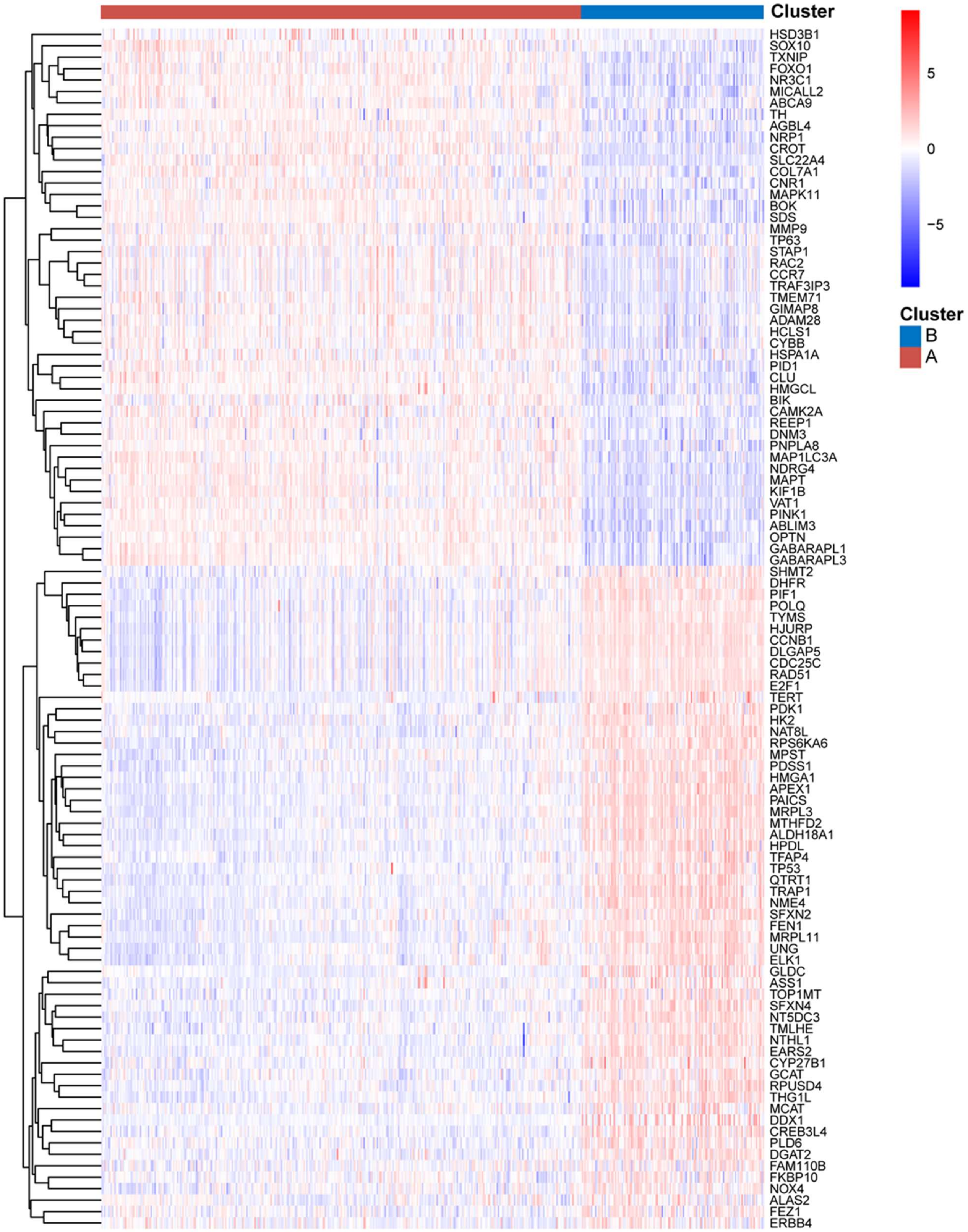

Cluster
HSD3B1
SOX10
TXNIP
FOXO1
NR3C1
MICALL2
ABCA9
TH
AGBL4
NRP1
CROT
SLC22A4
COL7A1
CNR1
MAPK11
BOK
SDS
MMP9
TP63
STAP1
RAC2
CCR7
TRAF3IP3
TMEM71
GIMAP8
ADAM28
HCLS1
CYBB
HSPA1A
PID1
CLU
HMGCL
BIK
CAMK2A
REEP1
DNM3
PNPLA8
MAP1LC3A
NDRG4
MAPT
KIF1B
VAT1
PINK1
ABLIM3
OPTN
GABARAPL1
GABARAPL3
SHMT2
DHFR
PIF1
POLQ
TYMS
HJURP
CCNB1
DLGAP5
CDC25C
RAD51
E2F1
TERT
PDK1
HK2
NAT8L
RPS6KA6
MPST
PDSS1
HMGA1
APEX1
PAICS
MRPL3
MTHFD2
ALDH18A1
HPDL
TFAP4
TP53
QTRT1
TRAP1
NME4
SFXN2
FEN1
MRPL11
UNG
ELK1
GLDC
ASS1
TOP1MT
SFXN4
NT5DC3
TMLHE
NTHL1
EARS2
CYP27B1
GCAT
RPUSD4
THG1L
MCAT
DDX1
CREB3L4
PLD6
DGAT2
FAM110B
FKBP10
NOX4
ALAS2
FEZ1
ERBB4
5
0
−5
Cluster
B
A

Supplementary Figure 1. Heatmap of MRGs expression in NB patients of GSE49710

Heatmap illustrating the expression levels of 105 MRGs across 2 distinct NB patient clusters identified in the GSE49710 dataset. MRG: mitochondria-related gene; NB: neuroblastoma.

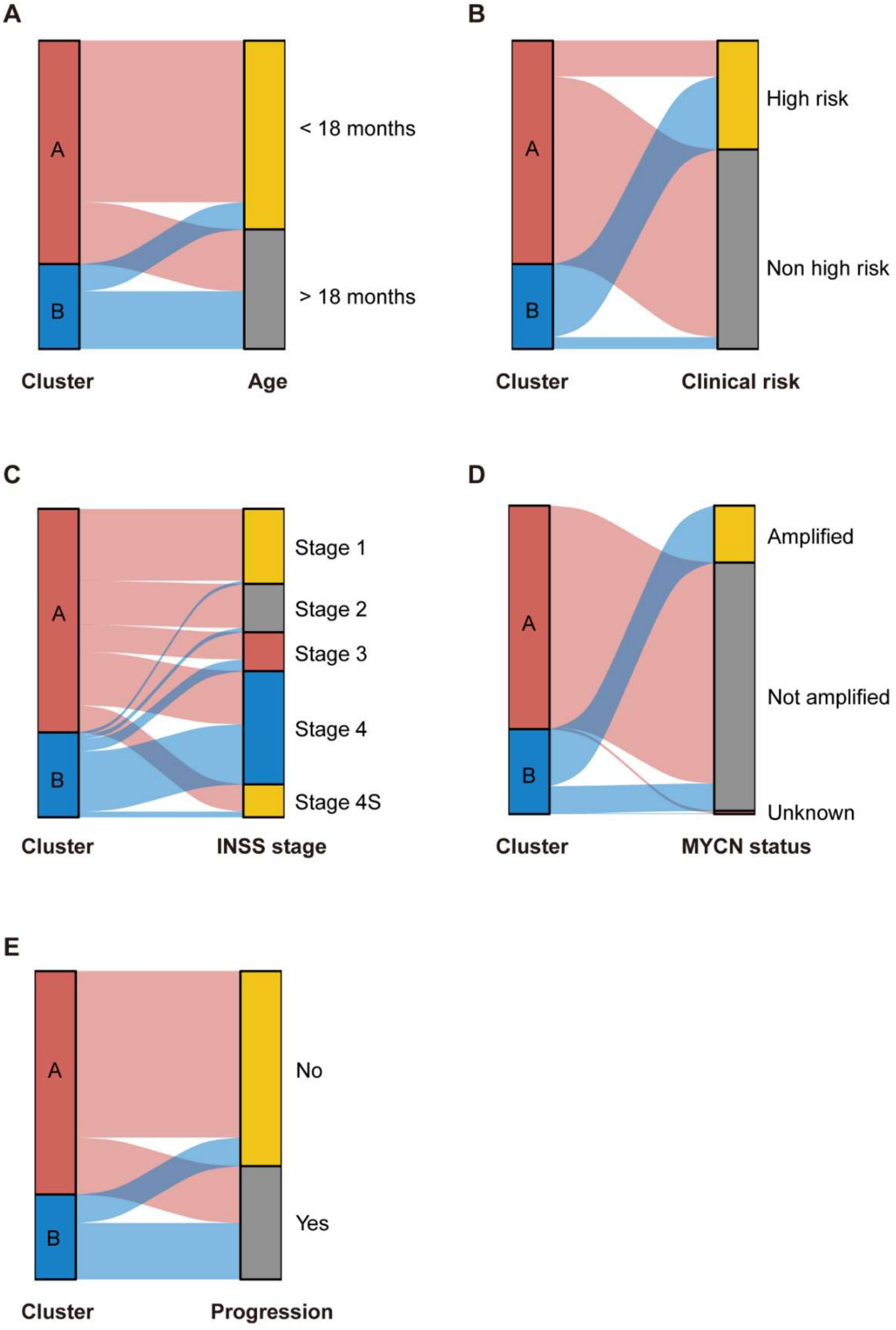
A
A
B
< 18 months
> 18 months
Cluster
Age
B
A
B
High risk
Non high risk
Cluster
Clinical risk
C
A
B
Stage 1
Stage 2
Stage 3
Stage 4
Stage 4S
Cluster
INSS stage
D
A
B
Amplified
Not amplified
Unknown
Cluster
MYCN status
E
A
B
No
Yes
Cluster
Progression

Supplementary Figure 2. Sankey diagrams reflecting the distribution of clinical characteristics across Clusters A and B of GSE49710

The Sankey diagrams displaying the distribution of patients between Cluster A and B by age (A), clinical risk categories (B), INSS stage (C), MYCN status (D), and progression status (E).

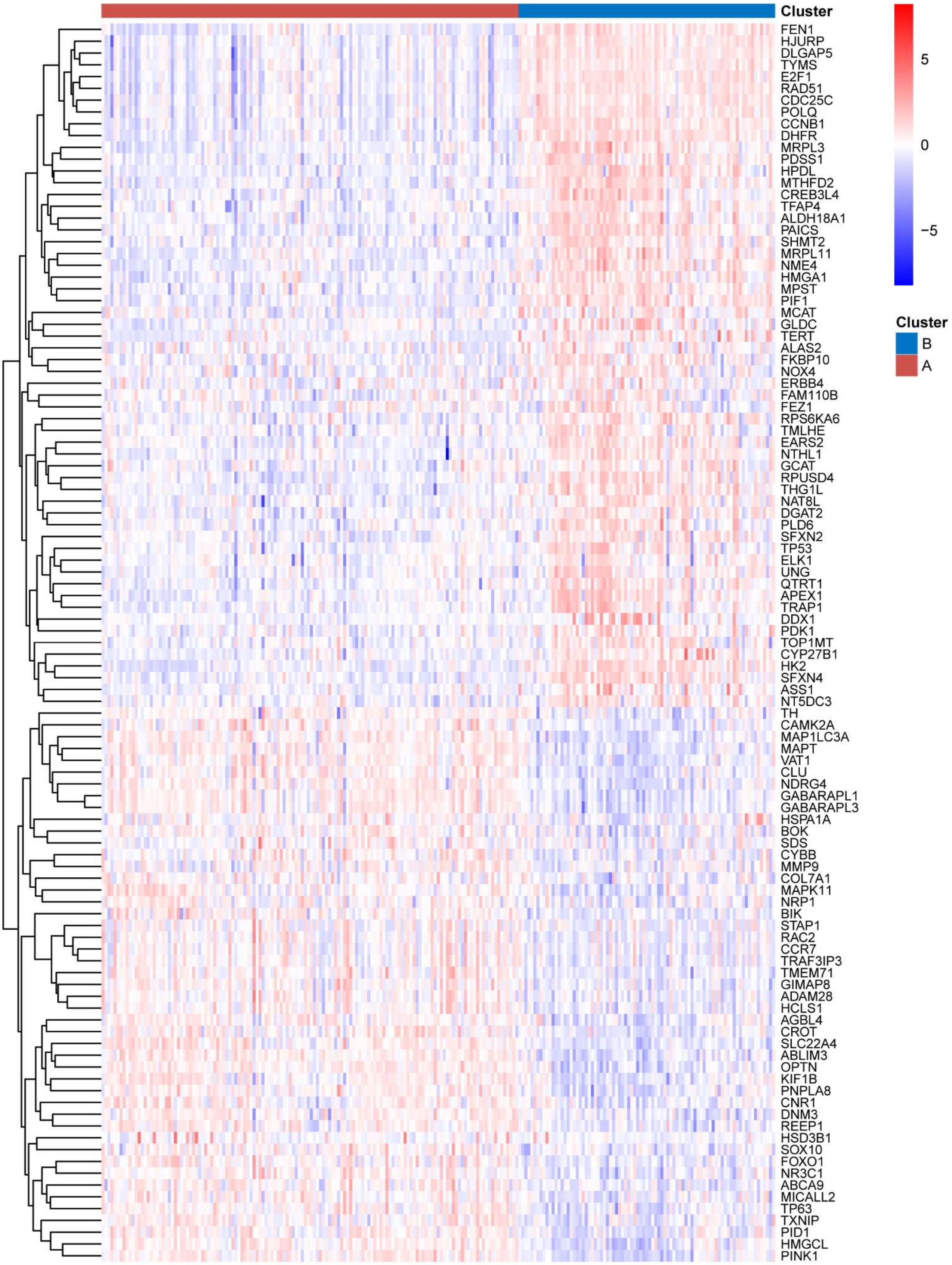
Cluster
FEN1
HJURP
DLGAP5
TYMS
E2F1
RAD51
CDC25C
POLQ
CCNB1
DHFR
MRPL3
PDSS1
HPDL
MTHFD2
CREB3L4
TFAP4
ALDH18A1
PAICS
SHMT2
MRPL11
NME4
HMGA1
MPST
PIF1
MCAT
GLDC
TERT
ALAS2
FKBP10
NOX4
ERBB4
FAM110B
FEZ1
RPS6KA6
TMLHE
EARS2
NTHL1
GCAT
RPUSD4
THG1L
NAT8L
DGAT2
PLD6
SFXN2
TP53
ELK1
UNG
QTRT1
APEX1
TRAP1
DDX1
PDK1
TOP1MT
CYP27B1
HK2
SFXN4
ASS1
NT5DC3
TH
CAMK2A
MAP1LC3A
MAPT
VAT1
CLU
NDRG4
GABARAPL1
GABARAPL3
HSPA1A
BOK
SDS
CYBB
MMP9
COL7A1
MAPK11
NRP1
BIK
STAP1
RAC2
CCR7
TRAF3IP3
TMEM71
GIMAP8
ADAM28
HCLS1
AGBL4
CROT
SLC22A4
ABLIM3
OPTN
KIF1B
PNPLA8
CNR1
DNM3
REEP1
HSD3B1
SOX10
FOXO1
NR3C1
ABCA9
MICALL2
TP63
TXNIP
PID1
HMGCL
PINK1
5
0
−5
Cluster
B
A

Supplementary Figure 3. Heatmap of MRGs expression in NB patients of E-MTAB-8248

Heatmap illustrating the expression levels of 105 MRGs across 2 distinct NB patient clusters identified in the E-MTAB-8248 dataset. MRG: mitochondria-related gene; NB: neuroblastoma.

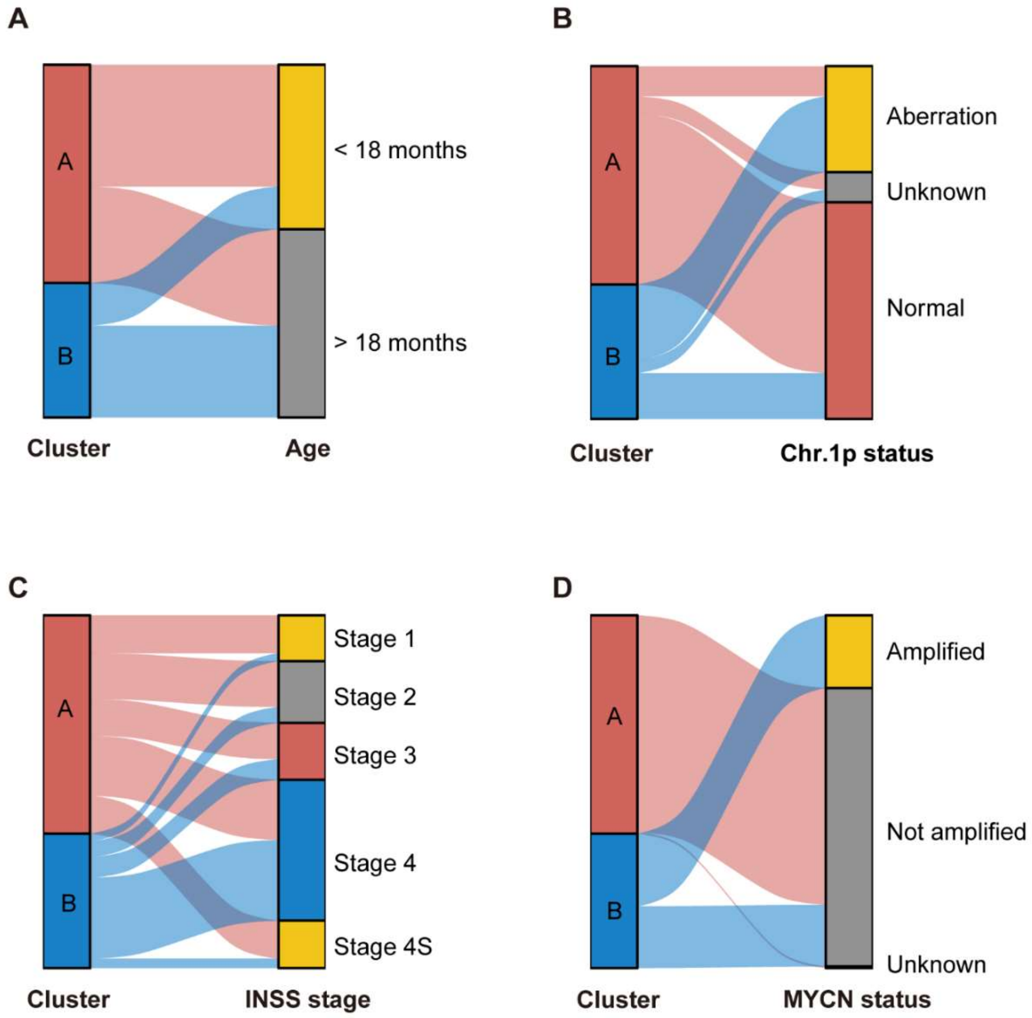

A
A
B
< 18 months
> 18 months
Cluster
Age
B
A
B
Aberration
Unknown
Normal
Cluster
Chr.1p status
C
A
B
Stage 1
Stage 2
Stage 3
Stage 4
Stage 4S
Cluster
INSS stage
D
A
B
Amplified
Not amplified
Unknown
Cluster
MYCN status

Supplementary Figure 4. Sankey diagrams reflecting the distribution of clinical characteristics across Clusters A and B of E-MTAB-8248

The Sankey diagrams displaying the distribution of patients between Cluster A and B by age (A), chromosome 1p status (B), INSS stage (C), and MYCN status (D). Chr: chromosome.

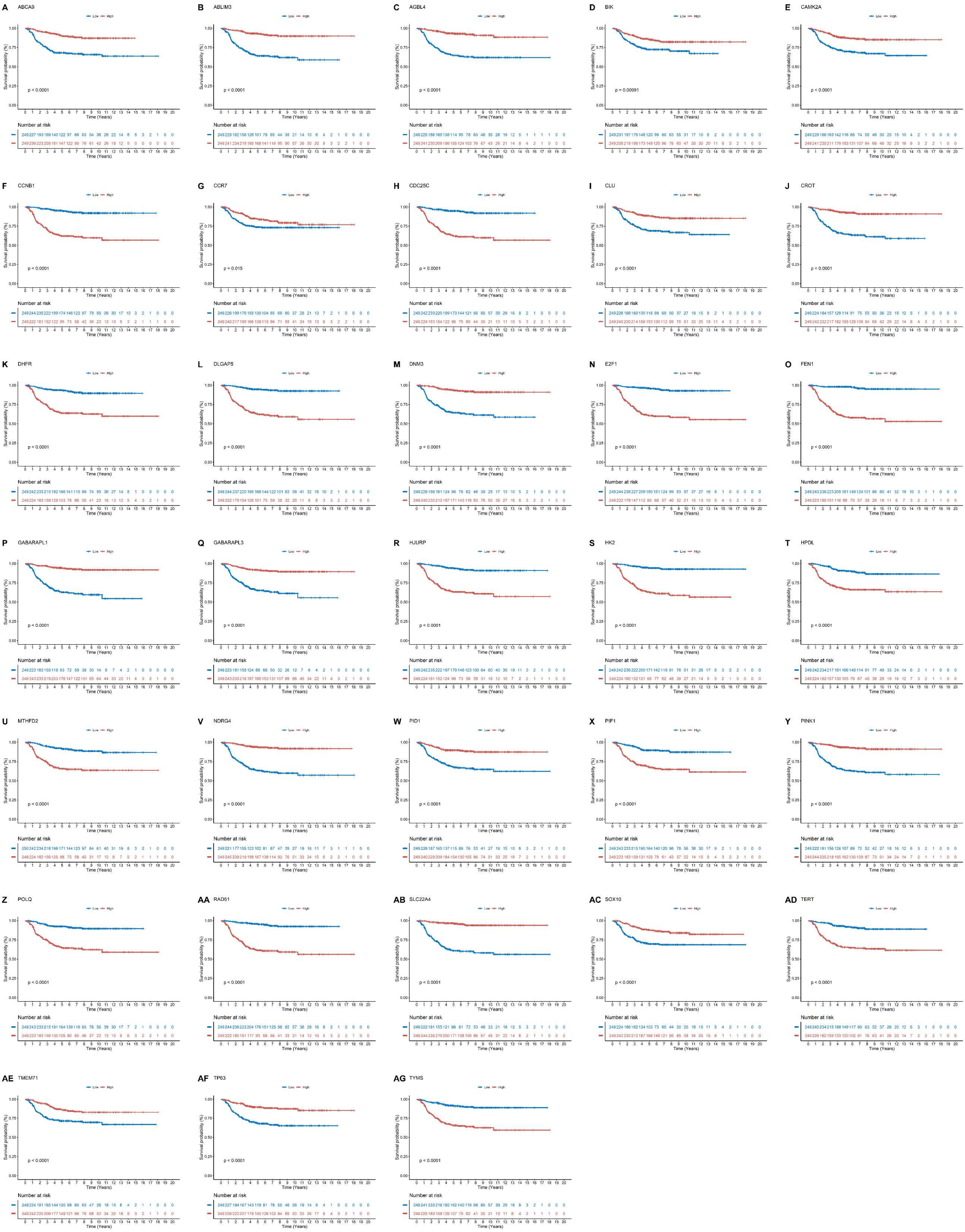

A ABCA9
B ABLIM3
C AGBL4
D BIK
E CAMK2A
F CCNB1
G CCR7
H CDC25C
I CLU
J CROT
K DHFR
L DLGAP5
M DNM3
N E2F1
O FEN1
P GABARAPL1
Q GABARAPL3
R HJURP
S HK2
T HPDL
U MTHFD2
V NDRG4
W PID1
X PIF1
Y PINK1
Z POLQ
AA RAD51
AB SLC22A4
AC SOX10
AD TERT
AE TMEM71
AF TP63
AG TYMS
Low
High
Survival probability (%)
Time (Years)
Number at risk
p < 0.0001
p = 0.00091
p = 0.015

Supplementary Figure 5. Kaplan-Meier survival curves for each of the 33 intersecting genes in the GSE49710 dataset

The collection of panels (A-AG) depicting Kaplan-Meier survival curves for patients stratified by the median expression levels of each of the 33 intersecting genes in the GSE49710 dataset. Each panel corresponds to one gene, with P values denoting the significance of difference in survival between the two groups.

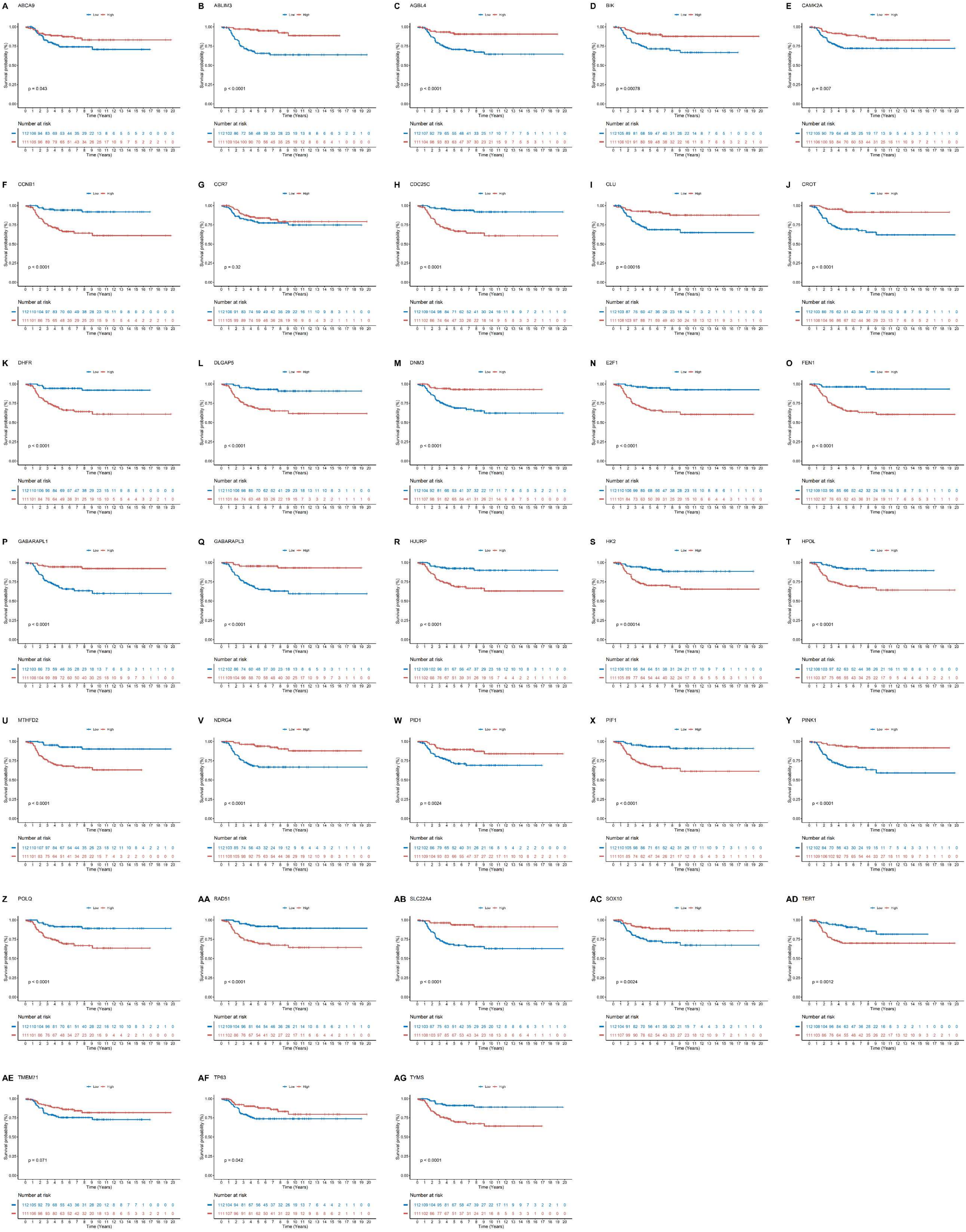

A ABCA9 p = 0.043
B ABLIM3 p < 0.0001
C AGBL4 p < 0.0001
D BIK p = 0.00078
E CAMK2A p = 0.007
F CCNB1 p < 0.0001
G CCR7 p = 0.32
H CDC25C p < 0.0001
I CLU p = 0.00016
J CROT p < 0.0001
K DHFR p < 0.0001
L DLGAP5 p < 0.0001
M DNM3 p < 0.0001
N E2F1 p < 0.0001
O FEN1 p < 0.0001
P GABARAPL1 p < 0.0001
Q GABARAPL3 p < 0.0001
R HJURP p < 0.0001
S HK2 p = 0.00014
T HPDL p < 0.0001
U MTHFD2 p < 0.0001
V NDRG4 p < 0.0001
W PID1 p = 0.0024
X PIF1 p < 0.0001
Y PINK1 p < 0.0001
Z POLQ p < 0.0001
AA RAD51 p < 0.0001
AB SLC22A4 p < 0.0001
AC SOX10 p = 0.0024
AD TERT p = 0.0012
AE TMEM71 p = 0.071
AF TP63 p = 0.042
AG TYMS p < 0.0001
Low High
Survival probability (%)
Time (Years)
Number at risk

Supplementary Figure 6. Kaplan-Meier survival curves for each of the 33 intersecting genes in the E-MTAB-8248 dataset

The collection of panels (A-AG) depicting Kaplan-Meier survival curves for patients stratified by the median expression levels of each of the 33 intersecting genes in the E-MTAB-824 dataset. Each panel corresponds to one gene, with P values denoting the significance of difference in survival between the two groups.

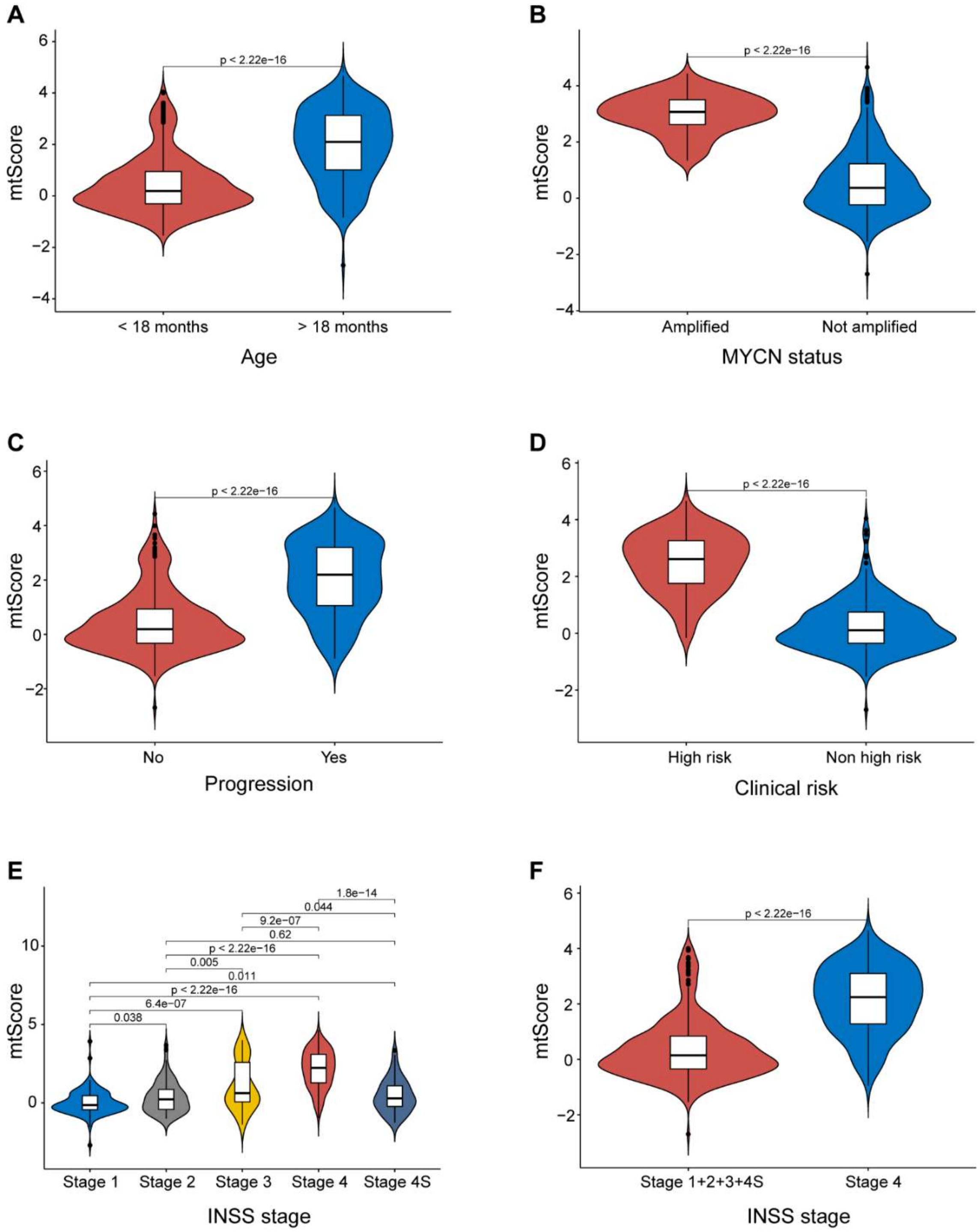
A
mtScore
p < 2.22e−16
< 18 months
> 18 months
Age
B
mtScore
p < 2.22e−16
Amplified
Not amplified
MYCN status
C
mtScore
p < 2.22e−16
No
Yes
Progression
D
mtScore
p < 2.22e−16
High risk
Non high risk
Clinical risk
E
mtScore
1.8e−14
0.044
9.2e−07
0.62
p < 2.22e−16
0.005
0.011
p < 2.22e−16
6.4e−07
0.038
Stage 1
Stage 2
Stage 3
Stage 4
Stage 4S
INSS stage
F
mtScore
p < 2.22e−16
Stage 1+2+3+4S
Stage 4
INSS stage

Supplementary Figure 7. Violin plots depicting the distribution of mtScores across clinical characteristics in the GSE49710 dataset

(A) Comparison of mtScores across different age groups. (B) Comparison of mtScores across different MYCN status groups. (C) Comparison of mtScores across different progression status groups. (D) Comparison of mtScores across different clinical risk groups. (E) Comparison of mtScores across different INSS stages groups. (F) Comparison of mtScores between INSS stage 4 and other INSS stages.

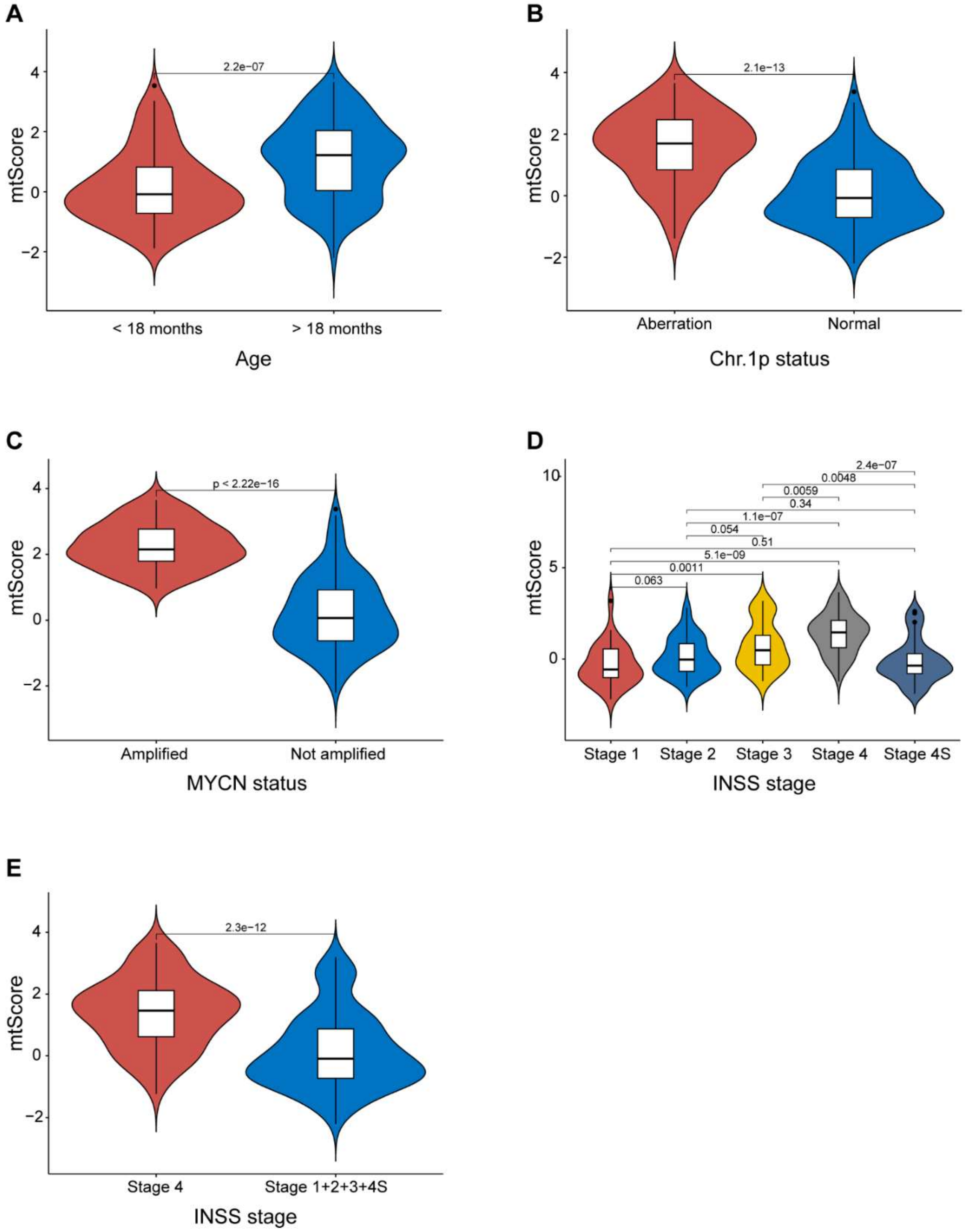

A
mtScore
4
2
0
−2
2.2e−07
< 18 months
> 18 months
Age
B
mtScore
2.1e−13
Aberration
Normal
Chr.1p status
C
mtScore
p < 2.22e−16
Amplified
Not amplified
MYCN status
D
mtScore
10
5
0
2.4e−07
0.0048
0.0059
0.34
1.1e−07
0.054
0.51
5.1e−09
0.0011
0.063
Stage 1
Stage 2
Stage 3
Stage 4
Stage 4S
INSS stage
E
mtScore
2.3e−12
Stage 4
Stage 1+2+3+4S
INSS stage

Supplementary Figure 8. Violin plots depicting the distribution of mtScores across clinical characteristics in the E-MTAB-8248 Dataset

(A) Comparison of mtScores across different age groups. (B) Comparison of mtScores across different chromosome 1p status groups. (C) Comparison of mtScores across different MYCN status groups. (D) Comparison of mtScores across different INSS stage groups. (E) Comparison of mtScores between INSS stage 4 and other INSS stages.

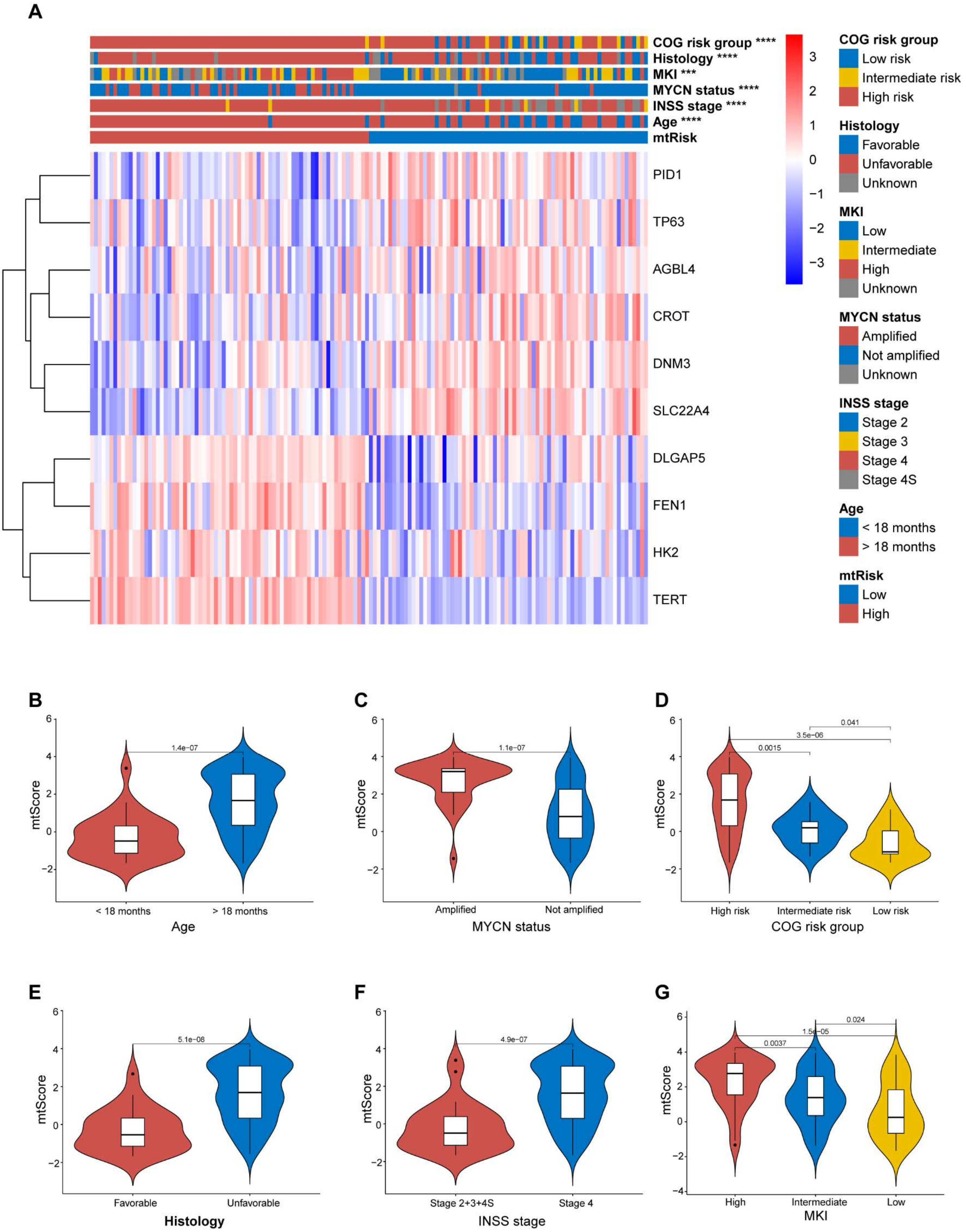
A
COG risk group ****
Histology ****
MKI ***
MYCN status ****
INSS stage ****
Age ****
mtRisk
PID1
TP63
AGBL4
CROT
DNM3
SLC22A4
DLGAP5
FEN1
HK2
TERT
3
2
1
0
−1
−2
−3
COG risk group
Low risk
Intermediate risk
High risk
Histology
Favorable
Unfavorable
Unknown
MKI
Low
Intermediate
High
Unknown
MYCN status
Amplified
Not amplified
Unknown
INSS stage
Stage 2
Stage 3
Stage 4
Stage 4S
Age
< 18 months
> 18 months
mtRisk
Low
High
B
mtScore
1.4e−07
< 18 months
> 18 months
Age
C
mtScore
1.1e−07
Amplified
Not amplified
MYCN status
D
mtScore
0.041
3.5e−06
0.0015
High risk
Intermediate risk
Low risk
COG risk group
E
mtScore
5.1e−08
Favorable
Unfavorable
Histology
F
mtScore
4.9e−07
Stage 2+3+4S
Stage 4
INSS stage
G
mtScore
0.024
1.5e−05
0.0037
High
Intermediate
Low
MKI

Supplementary Figure 9. Correlation of mtScore with clinical and molecular features in TARGET-NBL

(A) Heatmap depicting the distribution of 10 genes expression used to calculate mtScore stratified by high and low mtRisk, and the distribution of clinical features (COG risk group, histology, MKI, MYCN status, INSS stage, and age) stratified by high and low mtRisk. (B-G) Violin plots illustrating the distribution of mtScores in relation to various clinical factors: age groups (B), MYCN status (C), COG risk groups (D), histology (E), INSS stages (F), MKI classification (G). (***P<0.001, ****P<0.0001)

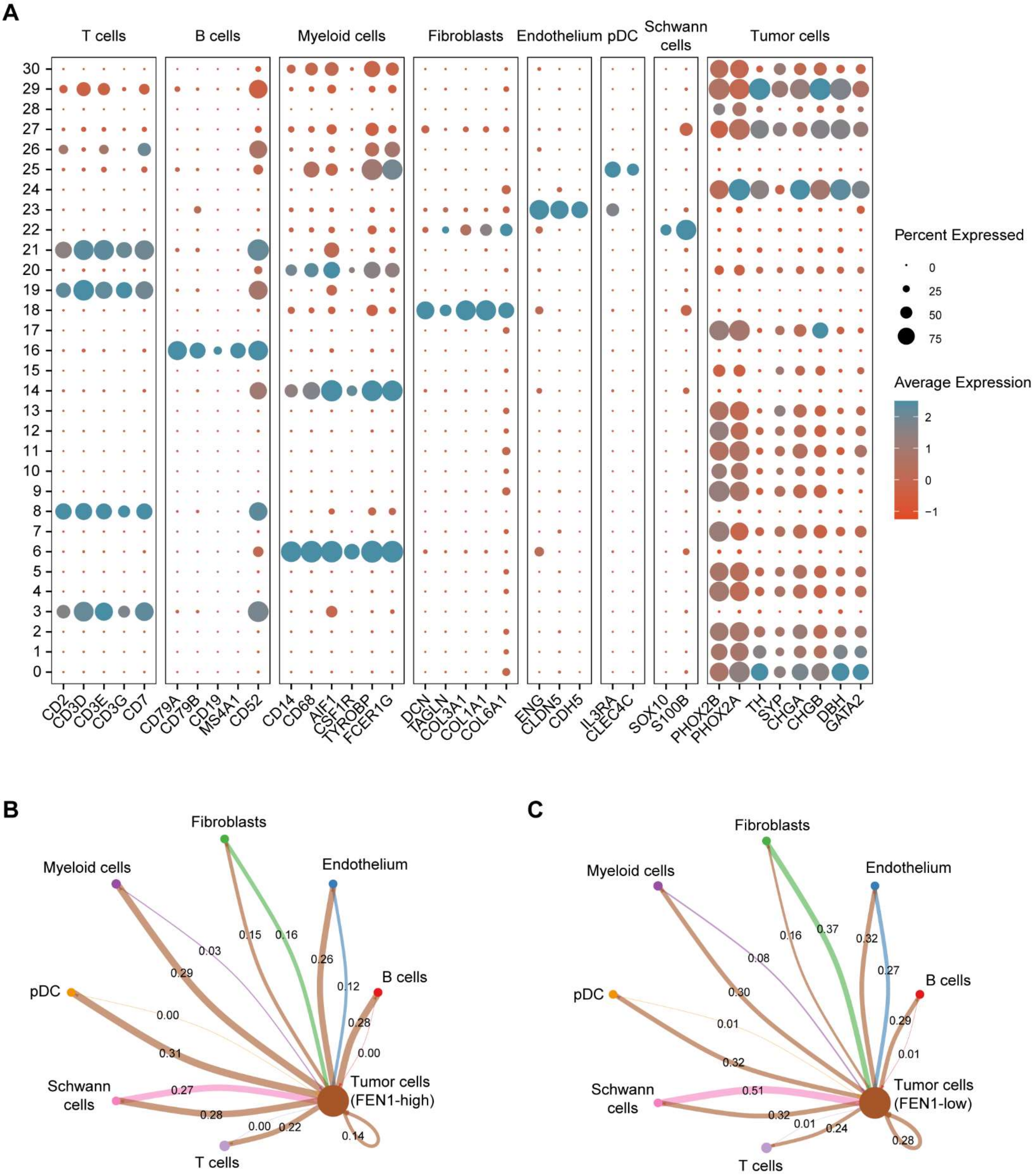
A
T cells
B cells
Myeloid cells
Fibroblasts
Endothelium
pDC
Schwann cells
Tumor cells
Percent Expressed
0
25
50
75
Average Expression
2
1
0
−1
CD2
CD3D
CD3E
CD3G
CD7
CD79A
CD79B
CD19
MS4A1
CD52
CD14
CD68
AIF1
CSF1R
TYROBP
FCER1G
DCN
TAGLN
COL3A1
COL1A1
COL6A1
ENG
CLDN5
CDH5
IL3RA
CLEC4C
SOX10
S100B
PHOX2B
PHOX2A
TH
SYP
CHGA
CHGB
DBH
GATA2
B
Fibroblasts
Myeloid cells
Endothelium
B cells
pDC
Schwann cells
T cells
Tumor cells (FEN1-high)
0.03
0.15
0.16
0.26
0.12
0.29
0.00
0.28
0.00
0.31
0.27
0.28
0.00
0.22
0.14
C
Fibroblasts
Myeloid cells
Endothelium
B cells
pDC
Schwann cells
T cells
Tumor cells (FEN1-low)
0.16
0.37
0.32
0.08
0.27
0.30
0.29
0.01
0.01
0.32
0.51
0.32
0.01
0.24
0.28

Supplementary Figure 10. Single-cell transcriptome sequencing data analysis to explore FEN1's essential role

(A) Dot plot of cell markers used in single-cell data annotation. (B) Cell-cell communication weights network of FEN1-high tumor cells with the surrounding microenvironment. (C) Cell-cell communication weights network of FEN1-low tumor cells with the surrounding microenvironment. pDC: plasmacytoid dendritic cell.
